# Discriminating prevalent type 2 diabetes among community-dwelling older adults with metabolic dysfunction-associated steatotic liver disease: a comparative analysis of 12 insulin resistance surrogates

**DOI:** 10.3389/fendo.2026.1846547

**Published:** 2026-07-08

**Authors:** Xianshang Zhu, Zengrui Wang, Yan Fang, Zong Ning, Xia Yang

**Affiliations:** 1 Department of General Medicine, The First Affiliated Hospital of Guangxi Medical University, Nanning, China; 2 Department of General Medicine, Gansu Provincial Hospital, Lanzhou, Gansu, China

**Keywords:** insulin resistance, metabolic dysfunction-associated steatotic liver disease, older adults, triglyceride-glucose index, type 2 diabetes mellitus

## Abstract

**Background:**

Metabolic dysfunction-associated steatotic liver disease frequently coexists with type 2 diabetes mellitus (T2DM). Because the gold standard for assessing insulin resistance (IR) is difficult to implement in primary care settings, this cross-sectional study aimed to compare the associations and discriminatory ability of 12 surrogate IR indexes for prevalent T2DM among community-dwelling older adults with metabolic dysfunction-associated fatty liver disease.

**Methods:**

This study was based on a health examination cohort from a community in northwestern China and included 2641 eligible participants, of whom 605 were older adults with metabolic dysfunction-associated steatotic liver disease. Multivariable logistic regression, receiver operating characteristic (ROC) curve analysis, restricted cubic spline (RCS) analysis, subgroup analysis, and exploratory internal model-discrimination analyses were performed to evaluate the associations and discriminatory ability of 12 surrogate IR indexes for prevalent T2DM.

**Results:**

After multivariable adjustment, TyG, TyG-ABSI, TyG-WWI, and TyG-WC were significantly associated with prevalent T2DM in participants with metabolic dysfunction-associated steatotic liver disease (all P < 0.05). TyG showed the highest discriminatory ability (AUC = 0.726, 95% CI: 0.686-0.766), with an optimal cut-off value of 9.225. After Bonferroni correction, it performed significantly better than the other indicators. Sensitivity analyses addressing the mathematical coupling between TyG and T2DM diagnostic criteria confirmed the robustness of these findings. Even after excluding T2DM cases defined solely by fasting plasma glucose, TyG remained the best-performing index (AUC = 0.707). RCS analyses showed significant overall associations but did not support statistically significant nonlinearity for TyG, TyG-ABSI, TyG-WWI, or TyG-WC (all P-nonlinear > 0.05). No significant interactions were observed in subgroup analyses.

**Conclusions:**

TyG, TyG-ABSI, TyG-WWI, and TyG-WC were associated with prevalent T2DM and showed moderate discriminatory ability. Sensitivity analyses addressing the shared FPG component confirmed the robustness of these associations. These findings support TyG as a simple, integrated metabolic marker for alerting to prevalent T2DM in this population.

## 1 Introduction

Metabolic dysfunction-associated steatotic liver disease (MASLD), formerly term-defined as non-alcoholic fatty liver disease (NAFLD) or metabolic dysfunction-associated fatty liver disease (MAFLD) under the newly updated international consensus (1), and type 2 diabetes mellitus (T2DM) are two common and closely interrelated metabolic disorders in older adults (2). With population ageing and the growing burden of metabolic diseases, the coexistence of MASLD and T2DM has become an important public health issue (3). Global studies have shown that the prevalence of MASLD is approximately 25.0% in the general population and as high as 50.7% among overweight and obese adults, while the pooled prevalence of T2DM in patients with MASLD is 19.7% (4). In China, several studies have demonstrated substantial population heterogeneity in the prevalence of MASLD, ranging from 22.75% to 35.58% in nationwide health examination populations (5) and reaching 40.3% among middle-aged and older adults in community settings (6). Notably, MASLD is closely associated with T2DM. The prevalence of diabetes among patients with MASLD has been reported to range from 25.0% to 33.9% (7, 8), whereas the prevalence of MASLD in patients with diabetes is as high as 53.8% (6). Moreover, the coexistence of T2DM in patients with MASLD is associated with a further increase in the risks of adverse outcomes, including cardiovascular disease, chronic kidney disease, and all-cause mortality (9). Early identification of T2DM in community-dwelling older adults with MASLD is therefore of considerable clinical and public health importance.

Insulin resistance (IR) is a key shared pathophysiological basis of both MASLD and T2DM. IR promotes hepatic lipid accumulation, aggravates lipid metabolic disorders, and impairs glucose utilization, thereby contributing to the development and progression of both MASLD and T2DM (3). At present, the hyperinsulinemic-euglycemic clamp is considered the gold standard for assessing IR; however, its complexity and high cost limit its widespread use in large-scale population studies and routine community screening (10). Although the homeostasis model assessment of insulin resistance (HOMA-IR) is also commonly used to evaluate IR, it still depends on insulin measurement, which restricts its application in primary care and routine health examinations (11). Therefore, the identification of simple, inexpensive, and reproducible surrogate markers of IR is of considerable value for the early detection of T2DM in community populations.

In recent years, a variety of surrogate indexes of IR derived from routine biochemical markers and anthropometric parameters have been widely used in studies of metabolic disorders, including the triglyceride-glucose index (TyG), TyG-body mass index (TyG-BMI), TyG-waist circumference (TyG-WC), the metabolic score for insulin resistance (METS-IR), TyG-waist-to-height ratio (TyG-WHtR), TyG-weight-adjusted waist index (TyG-WWI), TyG-a body shape index (TyG-ABSI), and the Chinese visceral adiposity index (CVAI) (12). These indexes are convenient to obtain, easy to calculate, and relatively inexpensive, and have been shown to be closely associated with T2DM, fatty liver disease, and other cardiometabolic abnormalities (13). However, existing studies have mainly focused on the general adult population or on single indexes. For the specific high-risk population of community-dwelling older adults with MASLD, there is still a lack of systematic comparisons of multiple surrogate IR indexes for identifying T2DM, and their relative discriminatory performance and clinical utility remain unclear.

Against this background, we conducted the present cross-sectional study based on a large community-based health examination cohort of older adults in northwestern China. We aimed to systematically compare the associations of 12 surrogate IR indexes with prevalent T2DM in older adults with MASLD and to compare their cross-sectional associations and discriminatory capacity for an already prevalent comorbidity with and without prevalent T2DM. We further examined the shape of these cross-sectional associations and conducted exploratory internal model-comparison analyses, with the goal of providing evidence on simple and routinely available metabolic indicators in this population.

## 2 Methods

### 2.1 Study design and study population

This was a community-based cross-sectional study. Data were retrospectively extracted from routine community health examination records in a community health service center in Gansu Province, northwestern China, and included older residents who underwent routine health examinations between April 2023 and March 2024. The source data were originally collected and managed in Gansu Province under the approved local protocol of Gansu Provincial People’s Hospital (approval no. 2024-353). The de-identified existing dataset was subsequently analyzed at the First Affiliated Hospital of Guangxi Medical University under a separate ethics approval for secondary analysis (NO: 2026-S0660).

The inclusion criteria were as follows: (1) age >= 65 years; (2) continuous residence in the community for at least 2 years; (3) availability of routine health-examination records and key clinical and laboratory variables; and (4) no history of severe liver disease (such as viral hepatitis or cirrhosis) or malignant tumors. Because this was a retrospective secondary analysis of de-identified routine health-examination records, informed consent was waived by the Ethics Committee of the First Affiliated Hospital of Guangxi Medical University for the current secondary analysis (NO: 2026-S0660).

The exclusion criteria were as follows: (1) missing, nonnumeric, or clinically implausible data on key variables, including FPG, TG, WC, BMI, and HDL-C; (2) a community hospital-confirmed record of severe psychiatric disorder; (3) excessive alcohol consumption (>=210 g/week for men and >=140 g/week for women); (4) comorbid severe chronic diseases, including stroke, COPD, malignant tumors, or creatinine-based renal insufficiency; and (5) refusal or administrative restriction for use of routine health-examination data. Severe psychiatric disorder was based on the community hospital-confirmed severe mental disorder field, and renal insufficiency was screened using serum creatinine from the source health-examination record.

### 2.2 Data collection and quality control

#### 2.2.1 Assessment of demographic characteristics and lifestyle factors

Demographic characteristics and lifestyle-related factors were collected by uniformly trained primary care physicians using a standardized questionnaire. Smoking was defined as smoking at least one cigarette per day for more than 1 year. To minimize the potential confounding effect of alcohol-related liver injury, only light-to-moderate drinkers were included; participants with alcohol intake of ≥210 g/week for men or ≥140 g/week for women were excluded at enrollment. Regular exercise was defined as moderate-intensity or higher physical activity performed at least three times per week for at least 30 min each time; participants who did not meet this criterion were considered physically inactive. In addition, previously diagnosed clinical conditions, including hypertension and diabetes, were recorded in detail.

#### 2.2.2 Anthropometric measurements and blood pressure assessment

Anthropometric measurements were performed by community general practitioners while the participants were wearing light clothing and no shoes. The measurements included height, weight, and WC, from which BMI and waist-to-height ratio were calculated. Blood pressure was measured in the right upper arm after the participant had rested in a seated position for at least 5 min, using an automated electronic sphygmomanometer (YE660D). Systolic blood pressure and diastolic blood pressure were measured three times, and the average values were used in the analysis.

#### 2.2.3 Laboratory measurements

After an overnight fast of at least 8 h, 5 mL of venous blood was collected from each participant. Blood samples were analyzed using an URIT-8460 automated chemistry analyzer. The laboratory measurements included FPG, TG, total cholesterol (TC), HDL-C, low-density lipoprotein cholesterol (LDL-C), and liver and renal function parameters.

#### 2.2.4 Abdominal ultrasonography

Abdominal ultrasonography was performed by specialist physicians using a fully digital color Doppler ultrasound system (BLS-X3). According to the diagnostic criteria for MASLD, hepatic steatosis was considered present when the ultrasound report explicitly indicated “fatty liver” (14). All ultrasound reports were independently reviewed by two investigators. In cases of disagreement, a senior physician made the final judgement to ensure consistency and accuracy in the assessment of fatty liver.

### 2.3 Definition of MASLD

MASLD was defined according to the 2023 multi-society Delphi consensus statement on metabolic dysfunction-associated steatotic liver disease (15). Hepatic steatosis was confirmed by abdominal ultrasonography suggestive of fatty liver. To reduce overlap between the operational definition of MASLD and T2DM status, glucose abnormality and T2DM-related criteria were excluded from the diagnostic definition of MASLD in the present study.

After excluding participants with excessive alcohol consumption (>=210 g/week for men and >=140 g/week for women), MASLD was defined as the presence of hepatic steatosis together with at least one of the following non-glycemic metabolic cardiovascular risk factors: (1) BMI >= 24.0 kg/m^2^, or WC >= 90 cm in men and >=85 cm in women, or excessive body fat; (2) TG >= 1.7 mmol/L or current use of lipid-lowering medications; (3) reduced HDL-C (<=1.0 mmol/L in men and <=1.3 mmol/L in women) or current use of lipid-lowering medications; or (4) blood pressure >=130/85 mmHg or current use of antihypertensive medications.

### 2.4 Definition of T2DM

T2DM status was defined according to internationally accepted diagnostic criteria (16). Participants were considered to have T2DM if they met any of the following criteria: (1) typical symptoms of diabetes with a random plasma glucose level >=11.1 mmol/L; (2) FPG >=7.0 mmol/L; (3) 2-h plasma glucose >=11.1 mmol/L during an oral glucose tolerance test (OGTT); (4) glycated hemoglobin (HbA1c) >=6.5%; or (5) a confirmed diagnosis of T2DM made by a secondary or higher-level medical institution. Participants were also considered to have T2DM if they were receiving oral hypoglycemic agents or insulin, as this implies a prior medical diagnosis.

### 2.5 Study outcome

The main dependent variable for the MASLD subgroup analysis was the presence of comorbid prevalent T2DM among participants who met the diagnostic criteria for MASLD. Because of the cross-sectional design, this variable should be interpreted as a co-prevalent disease status rather than an incident outcome (17).

### 2.6 Calculation of surrogate indexes of insulin resistance

Twelve commonly used surrogate indexes of insulin resistance were evaluated, including the triglyceride-glucose index (TyG), TyG-body mass index (TyG-BMI), TyG-waist circumference (TyG-WC), TyG-waist-to-height ratio (TyG-WHtR), TyG-weight-adjusted waist index (TyG-WWI), TyG-a body shape index (TyG-ABSI), the metabolic score for insulin resistance (METS-IR), the visceral adiposity index (VAI), lipid accumulation product (LAP), the triglyceride/high-density lipoprotein cholesterol ratio (TG/HDL-C), the Chinese visceral adiposity index (CVAI), and the atherogenic index of plasma (AIP). All indexes were calculated according to the standard formulas reported in previous studies, and the detailed formulas are presented in **Supplementary Table 1**.

### 2.7 Statistical analysis

Participants were classified into four groups according to disease status: the normal group, the MASLD-only group (MASLD without T2DM), the T2DM-only group, and the MASLD with T2DM group. Baseline characteristics were described and compared across these groups. In addition, among participants with MASLD, comparisons were further performed between the MASLD-only group and the MASLD with T2DM group.

Normally distributed continuous variables were expressed as Mean ± SD, and comparisons between groups were performed using the independent-samples t test or one-way analysis of variance. Non-normally distributed continuous variables were expressed as median (IQR), and comparisons were performed using the Kruskal-Wallis rank sum test or Wilcoxon rank-sum test. Categorical variables were expressed as n (%), and comparisons were made using Pearson’s Chi-squared test or Fisher’s exact test. To address potential limitations of conventional hypothesis testing (where P-values are highly sensitive to sample size), we calculated standardized mean differences (SMDs) to evaluate the balance and magnitude of differences in baseline characteristics between participants with and without T2DM. An SMD of less than 0.1 was considered to indicate a negligible difference, whereas an SMD ≥ 0.1 was used as the threshold to define a clinically meaningful imbalance between the two groups.

To evaluate the robustness of our primary findings against potential selection bias from missing data, we further compared the baseline characteristics between participants with complete data and those with missing data, using the same standardized mean difference (SMD) threshold of < 0.1 to determine whether the two groups were well-balanced.

Before statistical modeling, we also examined the consistency between recorded hypertension history and measured blood pressure, evaluated multicollinearity among the 12 surrogate IR indexes using Spearman correlation coefficients and variance inflation factors, and conducted sensitivity analyses using measured blood-pressure-defined hypertension.

Multivariable logistic regression analyses were performed to examine the associations between the 12 surrogate IR indexes and T2DM among participants with MASLD. Each index was categorized into quartiles, and the lowest quartile was used as the reference group. Odds ratios (ORs) and 95% confidence intervals (95% CIs) were calculated, and tests for trend were performed (P for trend). Three models were constructed. Model 1 was an unadjusted model. Model 2 was adjusted for age, gender, educational level, and marital status. Model 3 was further adjusted for smoking, alcohol drinking, physical exercise, history of hypertension, alanine aminotransferase (ALT), aspartate aminotransferase (AST), TC, LDL-C, and Cr in addition to the variables included in Model 2. To prevent multicollinearity, separate regression models were established for each TyG-related index. Additionally, within each model, collinearity diagnostics were performed for all covariates by calculating the variance inflation factor (VIF). The VIFs for all analyzed variables were less than 5, indicating the absence of any severe multicollinearity that would warrant corrective action.

For indexes with P for trend < 0.05 in the fully adjusted multivariable regression analyses, ROC curve analyses were performed to calculate the area under the curve (AUC) and 95% CIs for discriminating prevalent T2DM. Differences in AUCs between indexes were compared using the DeLong test, and the optimal cut-off values were determined according to the Youden index. Because pairwise comparisons of AUCs involved multiple testing, P values were adjusted using the Bonferroni method. Surrogate measures were not used as primary ROC candidates if they did not meet the prespecified model 3 trend criteria.

Restricted cubic spline (RCS) models were used to assess the shape of the cross-sectional associations between key indexes and prevalent T2DM among participants with MASLD. Four knots were used in the RCS models, with knot locations automatically determined according to the distribution of the independent variables using the rms package. Model 1 was unadjusted. Model 2 was adjusted for gender, age, marital status and educational level. Model 3 was further adjusted for alcohol drinking, smoking, history of hypertension, physical exercise, ALT, AST, TC, LDL-C, and Cr. Overall associations (P-overall) and nonlinearity (P-nonlinear) were tested using the anova function. If the nonlinear test was not statistically significant, then the two-piecewise regression and inflection point analyses were not further interpreted.

Subgroup analyses were conducted according to age, gender, educational level, marital status, physical exercise, smoking, alcohol drinking, and history of hypertension to evaluate the consistency and robustness of the associations between key indexes and T2DM among participants with MASLD.

To assess exploratory incremental discrimination within this cross-sectional dataset, a basic model including age, gender, education, hypertension, exercise, and alcohol drinking was first constructed. The selected superior surrogate IR indexes were then added separately to the basic model. Changes in AUC were compared across models using the DeLong test. These analyses were interpreted as internal comparisons of discriminatory capacity for prevalent T2DM, rather than prospective predictions of incident T2DM (18, 19).

Calibration curves, NRI, IDI, and decision curve analyses were moved out of the main evidentiary framework because the study did not include longitudinal follow-up or incident T2DM outcomes. As a limited sensitivity check of internal probability agreement, the Brier score was calculated for exploratory logistic models.

All statistical analyses were performed using SPSS version 26.0 and R version 4.5.2. RCS analyses were conducted using the rms package, figures were generated using the ggplot2 package, and DCA was performed using the dcurves package. All statistical tests were two-sided, and P < 0.05 was considered statistically significant. Bonferroni correction was applied where appropriate for multiple comparisons.

## 3 Results

### 3.1 Participant selection and baseline characteristics of the overall cohort

**Figure 1:**
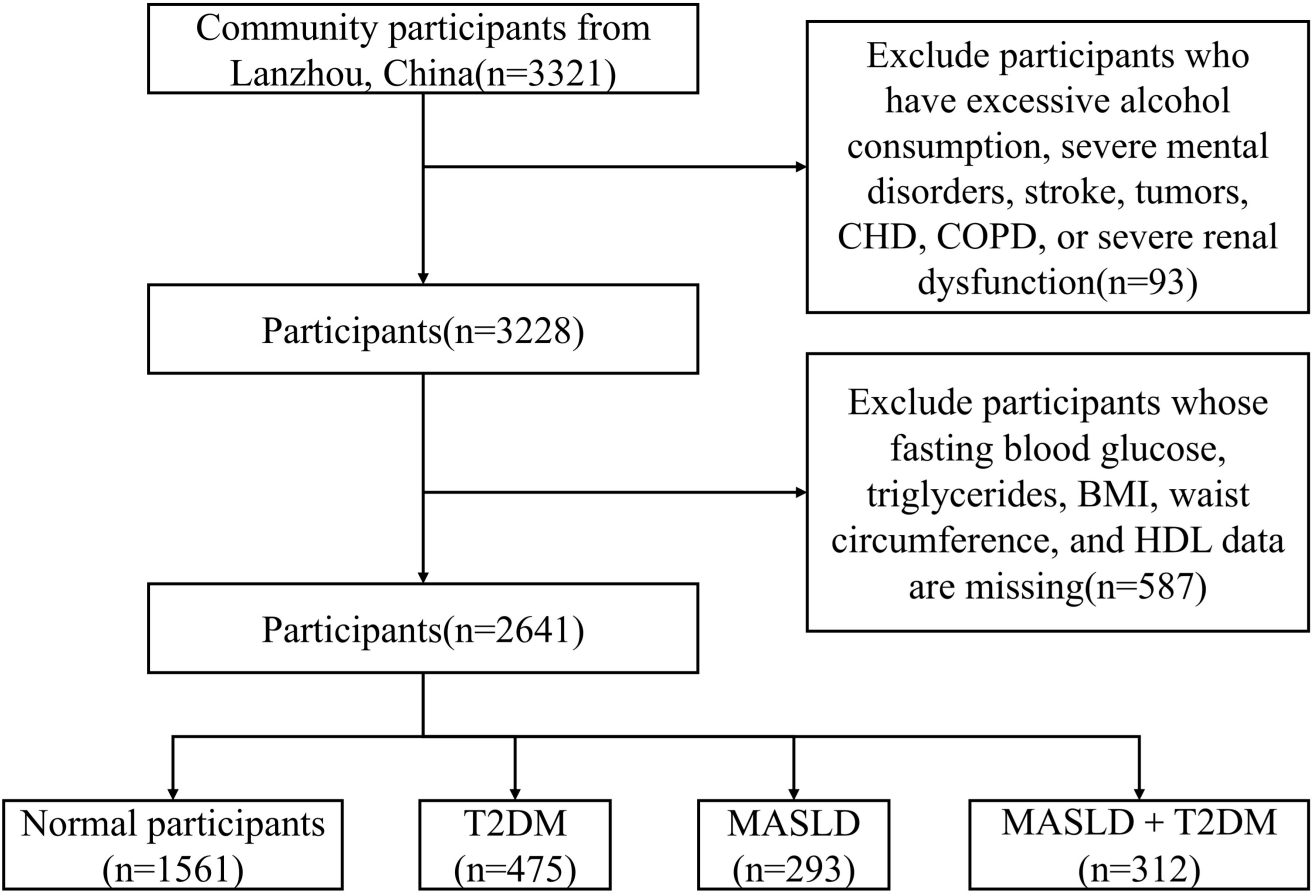
Flow chart of participant screening.

The flow chart of participant selection is shown in **Figure 1**. A total of 3321 adults aged 65 years or older who underwent routine health examinations were initially identified. Of these, 93 participants were excluded because of excessive alcohol consumption, severe psychiatric disorders, stroke, malignant tumors, coronary heart disease, chronic obstructive pulmonary disease (COPD), or severe renal insufficiency. A further 587 were excluded because of missing data on key variables, including fasting plasma glucose (FPG), triglycerides (TG), waist circumference (WC), body mass index (BMI), and high-density lipoprotein cholesterol (HDL-C). Finally, 2641 participants were included in the present study.

Exclusions of key variables during the data cleaning process reflected missing-coded, non-numeric, clinically implausible, or clinically disqualified records. Consequently, the prespecified complete-case analysis was retained, with the raw-data audit serving solely as supplementary sensitivity support rather than as a substitute for the analytical population.

According to the baseline status of MASLD and T2DM, the overall cohort was divided into four groups: the normal control group (n = 1561), the T2DM-only group (n = 475), the MASLD-only group (n = 293), and the MASLD with T2DM group (n = 312). The baseline characteristics of the overall study population are presented in **Supplementary Table 2**.

### 3.2 Baseline characteristics of participants with MASLD according to T2DM status

The main analytical population of this study consisted of 605 participants with MASLD, including 293 in the MASLD-only group and 312 in the MASLD with T2DM group. Their baseline characteristics are presented in **Table 1**. Age did not differ significantly between the MASLD with T2DM and MASLD-only groups (71.22 ± 5.42 vs. 71.83 ± 5.81 years, P = 0.192); however, the prevalence of hypertension was significantly higher in the former group (67% vs. 52%, P < 0.001). No significant differences were observed between the two groups regarding gender, educational level, marital status, physical exercise, smoking, or alcohol drinking (all P > 0.05).

**Table 1:** Comparison of baseline characteristics and insulin resistance indices between community-dwelling elderly MASLD patients with and without type 2 diabetes mellitus (N = 605).

Characteristics	MASLD (N = 293)^1^	MASLD+T2DM (N = 312)^1^	P^2^	SMD
Demographics and lifestyles				
Age	71.22 ± 5.42	71.83 ± 5.81	0.192	0.109
Gender			0.146	0.118
Male	119 (41%)	145 (46%)		
Female	174 (59%)	167 (54%)		
Education			0.119	1.127
Primary	54 (18%)	43 (14%)		
Junior high	239 (82%)	269 (86%)		
Marital			0.755	0.025
Unmarried	17 (5.8%)	20 (6.4%)		
Married	276 (94%)	292 (94%)		
Exercise			0.596	0.093
No	42 (14%)	35 (11%)		
Yes	251 (86%)	277 (89%)		
Smoke			0.825	0.018
No	246 (84%)	264 (85%)		
Yes	47 (16%)	48 (15%)		
Alcohol			0.690	0.032
No	260 (89%)	280 (90%)		
Yes	33 (11%)	32 (10%)		
Hypertension			<0.001	0.318
Yes	152 (52%)	210 (67%)		
No	141 (48%)	102 (33%)		
Drugs(Lowering blood glucose)			<0.001	1.293
No	293 (100%)	170 (54%)		
Yes	0 (0%)	142 (46%)		
Drugs(Lowering Blood pressure)			0.002	0.254
No	157 (54%)	128 (41%)		
Yes	136 (46%)	184 (59%)		
SBP (mmHg)	140.70 ± 15.69	143.22 ± 15.89	0.031	0.16
DBP (mmHg)	77.33 ± 10.83	79.31 ± 10.33	0.028	0.187
Height (cm)	162.75 ± 8.34	163.57 ± 8.80	0.316	0.096
Weight (kg)	68.23 ± 10.28	70.08 ± 11.11	0.109	0.172
WC (cm)	88.23 ± 9.88	89.62 ± 8.73	0.123	0.149
Laboratory tests				
BMI (kg/m2)	25.70 ± 2.85	26.12 ± 3.02	0.238	0.145
FPG (mmol/L)	5.32 ± 0.48	8.04 ± 2.47	<0.001	1.528
TC (mmol/L)	5.44 ± 1.32	5.56 ± 1.44	0.251	0.087
TG (mmol/L)	2.01 ± 1.01	2.30 ± 1.58	0.061	0.216
LDL (mmol/L)	2.53 ± 0.81	2.45 ± 0.84	0.369	0.101
HDL (mmol/L)	1.44 ± 0.41	1.40 ± 0.32	0.429	0.101
ALT (U/L)	27.77 ± 18.37	29.72 ± 19.69	0.093	0.102
AST (U/L)	25.67 ± 12.71	26.59 ± 13.40	0.250	0.071
Cr (µmol/L)	75.97 ± 20.25	77.12 ± 21.90	0.447	0.055
Surrogate IR indices				
TyG	8.94 ± 0.48	9.40 ± 0.63	<0.001	0.831
TyG-BMI	229.62 ± 28.10	245.58 ± 32.29	<0.001	0.527
TyG-WC	788.68 ± 99.38	842.51 ± 97.81	<0.001	0.546
TyG-WHtR	4.85 ± 0.62	5.16 ± 0.61	<0.001	0.496
TyG-WWI	95.81 ± 10.43	101.08 ± 10.22	<0.001	0.511
TyG-ABSI	7.11 ± 0.73	7.50 ± 0.73	<0.001	0.534
METS-IR	38.09 ± 5.21	40.62 ± 5.41	<0.001	0.477
CVAI	-595.06 ± 220.68	-575.85 ± 170.32	0.721	0.097
AIP	0.11 ± 0.25	0.16 ± 0.27	0.079	0.180
TG/HDL	3.49 ± 2.22	4.09 ± 3.31	0.079	0.213
VAI	5.82 ± 3.88	6.74 ± 5.97	0.227	0.182
LAP	55.38 ± 35.78	65.04 ± 47.70	0.009	0.229

^1^ Mean ± SD; n (%).

^2^ Wilcoxon rank sum test; Pearson’s Chi-squared test.

Data are expressed as mean ± SD for normally distributed continuous variables, median [IQR] for non-normally distributed continuous variables, and n (%) for categorical variables. SMD was calculated to assess the magnitude of differences in baseline characteristics between the MASLD and MASLD + T2DM groups. An SMD ≥ 0.1 indicates a meaningful clinical imbalance/difference between the two groups.

Beyond conventional statistical testing, SMD analysis provided a robust quantification of group differences. The two groups were well-balanced in terms of demographic and lifestyle characteristics, such as marital status (SMD = 0.025), smoking status (SMD = 0.018), and alcohol consumption (SMD = 0.032) (all SMDs < 0.1). Conversely, prominent clinical imbalances (SMDs ≥ 0.1) were observed in glucose metabolism and adiposity-related indices.

Among the 12 surrogate IR indexes, TyG, TyG-BMI, TyG-WC, TyG-WHtR, TyG-WWI, TyG-ABSI, and METS-IR were significantly elevated in the MASLD with T2DM group compared with the MASLD-only group (all P < 0.001). Additionally, a significant difference was observed for lipid accumulation product (LAP) (P = 0.009). Conversely, no significant differences were found regarding CVAI, AIP, TG/HDL-C, or VAI (all P > 0.05).

### 3.3 Associations of 12 surrogate IR indexes with T2DM among participants with MASLD

The results of the multivariable logistic regression analyses are shown in **Table 2**. In the fully adjusted model (Model 3), TyG, TyG-BMI, TyG-WC, TyG-WHtR, TyG-WWI, TyG-ABSI, and METS-IR were all significantly and positively associated with T2DM among participants with MASLD, with significant trends across quartiles (all P for trend < 0.05). Multicollinearity diagnostics showed that the Variance Inflation Factor (VIF) for all included variables in each model was below 5 (range: 1.03–3.19), indicating no serious multicollinearity that would affect the stability of regression coefficients. Detailed VIF values for each model are presented in **Supplementary Table 4**.

**Table 2:** Multivariable Logistic regression analysis of the association between 12 surrogate insulin resistance indices (categorized by quartiles) and the risk of type 2 diabetes mellitus in patients with MASLD.

Characteristic	Model 1		Model 2		Model 3	
	OR (95%CI)	P	OR (95%CI)	P	OR (95%CI)	P
TyG						
Continuous	4.800(3.409, 6.903)	<0.001	5.090(3.587, 7.388)	<0.001	5.423(3.737, 8.067)	<0.001
Q1	—		—		—	
Q2	1.718(1.066, 2.788)	0.0270	1.817(1.121, 2.969)	0.016	1.803(1.090, 3.003)	0.022
Q3	3.446(2.148, 5.597)	<0.001	3.701(2.282, 6.084)	<0.001	3.825(2.276, 6.525)	<0.001
Q4	10.22(6.071, 17.67)	<0.001	11.12(6.537, 19.42)	<0.001	11.79(6.714, 21.31)	<0.001
P for trend	2.133(1.816, 2.520)	<0.001	2.193(1.861, 2.603)	<0.001	2.254(1.890, 2.710)	<0.001
TyG-BMI						
Continuous	1.018(1.012, 1.024)	<0.001	1.018(1.013, 1.024)	<0.001	1.017(1.011, 1.023)	<0.001
Q1	—		—		—	
Q2	2.020(1.271, 3.234)	0.003	2.097(1.313, 3.377)	0.002	1.999(1.230, 3.273)	0.005
Q3	3.017(1.895, 4.855)	<0.001	3.027(1.893, 4.892)	<0.001	2.938(1.797, 4.856)	<0.001
Q4	4.122(2.568, 6.702)	<0.001	4.356(2.698, 7.133)	<0.001	3.921(2.363, 6.599)	<0.001
P for trend	1.591(1.371, 1.853)	<0.001	1.612(1.387, 1.880)	<0.001	1.564(1.333, 1.842)	<0.001
TyG-WC						
Continuous	1.006(1.004, 1.008)	<0.001	1.006(1.004, 1.008)	<0.001	1.005(1.004, 1.007)	<0.001
Q1	—		—		—	
Q2	1.954(1.232, 3.120)	0.005	1.932(1.216, 3.089)	0.006	1.746(1.080, 2.839)	0.024
Q3	2.354(1.484, 3.762)	<0.001	2.339(1.470, 3.752)	<0.001	2.111(1.298, 3.456)	0.003
Q4	4.382(2.724, 7.147)	<0.001	4.397(2.693, 7.282)	<0.001	4.074(2.428, 6.933)	<0.001
P for trend	1.582(1.363, 1.842)	<0.001	1.584(1.359, 1.853)	<0.001	1.546(1.314, 1.825)	<0.001
TyG-WHtR						
Continuous	2.301(1.742, 3.076)	<0.001	2.467(1.849, 3.332)	<0.001	2.343(1.725, 3.219)	<0.001
Q1	—		—		—	
Q2	1.407(0.888, 2.236)	0.147	1.480(0.930, 2.364)	0.099	1.347(0.828, 2.197)	0.231
Q3	2.602(1.643, 4.157)	<0.001	2.638(1.656, 4.240)	<0.001	2.468(1.512, 4.061)	<0.001
Q4	3.458(2.167, 5.581)	<0.001	3.855(2.376, 6.337)	<0.001	3.418(2.054, 5.757)	<0.001
P for trend	1.546(1.333, 1.798)	<0.001	1.591(1.365, 1.860)	<0.001	1.540(1.310, 1.815)	<0.001
TyG-WWI						
Continuous	1.055(1.037, 1.074)	<0.001	1.061(1.042, 1.081)	<0.001	1.059(1.039, 1.080)	<0.001
Q1	—		—		—	
Q2	1.848(1.166, 2.946)	0.009	1.901(1.194, 3.047)	0.007	1.862(1.147, 3.041)	0.012
Q3	2.001(1.263, 3.191)	0.003	2.158(1.349, 3.479)	0.001	2.064(1.266, 3.389)	0.004
Q4	4.991(3.083, 8.206)	<0.001	5.644(3.414, 9.496)	<0.001	5.361(3.169, 9.225)	<0.001
P for trend	1.620(1.395, 1.888)	<0.001	1.687(1.443, 1.982)	<0.001	1.657(1.407, 1.960)	<0.001
TyG-ABSI						
Continuous	2.215(1.730, 2.871)	<0.001	2.208(1.722, 2.866)	<0.001	2.190(1.691, 2.872)	<0.001
Q1	—		—		—	
Q2	2.061(1.300, 3.290)	0.002	2.041(1.282, 3.271)	0.003	1.995(1.231, 3.256)	0.005
Q3	1.853(1.168, 2.958)	0.009	1.806(1.135, 2.889)	0.013	1.731(1.068, 2.821)	0.027
Q4	5.496(3.379, 9.088)	<0.001	5.442(3.326, 9.054)	<0.001	5.325(3.187, 9.049)	<0.001
P for trend	1.630(1.403, 1.899)	<0.001	1.620(1.392, 1.892)	<0.001	1.607(1.372, 1.891)	<0.001
METS-IR						
Continuous	1.095(1.061, 1.131)	<0.001	1.096(1.061, 1.132)	<0.001	1.090(1.054, 1.128)	<0.001
Q1	—		—		—	
Q2	2.061(1.300, 3.290)	0.002	2.167(1.360, 3.480)	0.001	2.098(1.297, 3.418)	0.003
Q3	2.922(1.838, 4.691)	<0.001	2.862(1.789, 4.622)	<0.001	2.658(1.635, 4.362)	<0.001
Q4	3.266(2.049, 5.260)	<0.001	3.358(2.093, 5.450)	<0.001	3.083(1.888, 5.092)	<0.001
P for trend	1.476(1.274, 1.714)	<0.001	1.476(1.272, 1.718)	<0.001	1.434(1.228, 1.681)	<0.001
TG/HDL						
Continuous	1.082(1.020, 1.153)	0.011	1.085(1.022, 1.156)	0.010	1.088(1.023, 1.163)	0.009
Q1	—		—		—	
Q2	1.111(0.708, 1.745)	0.646	1.153(0.732, 1.818)	0.540	1.157(0.721, 1.859)	0.547
Q3	1.339(0.853, 2.109)	0.206	1.363(0.863, 2.158)	0.185	1.371(0.849, 2.219)	0.197
Q4	1.469(0.935, 2.315)	0.096	1.518(0.962, 2.404)	0.074	1.465(0.904, 2.385)	0.122
P for trend	1.144(0.991, 1.320)	0.066	1.153(0.998, 1.333)	0.054	1.141(0.979, 1.330)	0.092
VAI						
Continuous	1.038(1.005, 1.076)	0.030	1.049(1.013, 1.088)	0.009	1.050(1.013, 1.091)	0.010
Q1	—		—		—	
Q2	1.157(0.737, 1.818)	0.527	1.253(0.792, 1.989)	0.336	1.228(0.761, 1.986)	0.401
Q3	1.013(0.645, 1.590)	0.955	1.138(0.713, 1.819)	0.588	1.112(0.680, 1.822)	0.672
Q4	1.220(0.777, 1.918)	0.388	1.374(0.855, 2.217)	0.191	1.337(0.808, 2.218)	0.259
P for trend	1.048(0.908, 1.208)	0.524	1.089(0.937, 1.267)	0.268	1.081(0.920, 1.270)	0.345
CVAI						
Continuous	1.001(1.000, 1.001)	0.231	1.001(1.000, 1.002)	0.096	1.001(1.000, 1.002)	0.042
Q1	—		—		—	
Q2	1.469(0.935, 2.315)	0.096	1.632(1.030, 2.597)	0.038	1.687(1.043, 2.743)	0.034
Q3	1.356(0.864, 2.134)	0.187	1.523(0.956, 2.436)	0.077	1.715(1.049, 2.819)	0.032
Q4	1.096(0.698, 1.723)	0.689	1.225(0.767, 1.960)	0.396	1.382(0.824, 2.333)	0.223
P for trend	1.020(0.885, 1.177)	0.784	1.053(0.908, 1.223)	0.492	1.102(0.935, 1.302)	0.248
LAP						
Continuous	1.006(1.002, 1.010)	0.006	1.006(1.002, 1.011)	0.004	1.006(1.001, 1.010)	0.012
Q1	—		—		—	
Q2	1.219(0.776, 1.917)	0.390	1.318(0.833, 2.091)	0.240	1.275(0.789, 2.064)	0.322
Q3	1.219(0.776, 1.917)	0.390	1.278(0.807, 2.028)	0.297	1.093(0.670, 1.783)	0.721
Q4	1.824(1.158, 2.888)	0.010	1.975(1.240, 3.166)	0.004	1.731(1.049, 2.870)	0.032
P for trend	1.197(1.037, 1.383)	0.014	1.222(1.055, 1.417)	0.008	1.161(0.991, 1.361)	0.065
AIP						
Continuous	2.012(1.080, 3.789)	0.029	2.073(1.106, 3.929)	0.024	2.096(1.076, 4.130)	0.031
Q1	—		—		—	
Q2	1.111(0.708, 1.745)	0.646	1.153(0.732, 1.818)	0.540	1.157(0.721, 1.859)	0.547
Q3	1.339(0.853, 2.109)	0.206	1.363(0.863, 2.158)	0.185	1.371(0.849, 2.219)	0.197
Q4	1.469(0.935, 2.315)	0.096	1.518(0.962, 2.404)	0.074	1.465(0.904, 2.385)	0.122
P for trend	1.144(0.991, 1.320)	0.066	1.153(0.998, 1.333)	0.054	1.141(0.979, 1.330)	0.092

CI, confidence interval; OR, odds ratio. Model 1: No adjustment for any of the covariates. Model 2: Adjusted for age, gender, education level, and marital status. Model 3: Adjusted for variables in Model 2 and further adjusted for smoking, alcohol consumption, exercise, history of hypertension, ALT, AST, TC, LDL, and Cr.

Compared with the lowest quartile (Q1), the odds ratio (OR) for T2DM in the highest quartile (Q4) of TyG was 11.79 (95% CI: 6.714–21.31). The corresponding ORs for Q4 of TyG-BMI, TyG-WC, TyG-WHtR, TyG-WWI, TyG-ABSI, and METS-IR were 3.921 (95% CI: 2.363–6.599), 4.074 (95% CI: 2.428–6.933), 3.418 (95% CI: 2.054–5.757), 5.361 (95% CI: 3.169–9.225), 5.325 (95% CI: 3.187–9.049), and 3.083 (95% CI: 1.888–5.092).

No significant associations were observed for TG/HDL-C, VAI, CVAI, or AIP in any of the models. After fully adjusting for covariates, the trend association of LAP with T2DM was also no longer statistically significant. These negative findings suggest that indices incorporating broader adiposity or lipid components may provide limited independent information after adjustment for demographic, lifestyle, liver enzyme, and lipid covariates in this older MASLD population. Therefore, the primary ROC analyses were restricted to indices meeting the prespecified Model 3 P for trend criterion.

### 3.4 ROC curve analyses of surrogate IR indexes for discriminating prevalent T2DM among participants with MASLD

**Figure 2:**
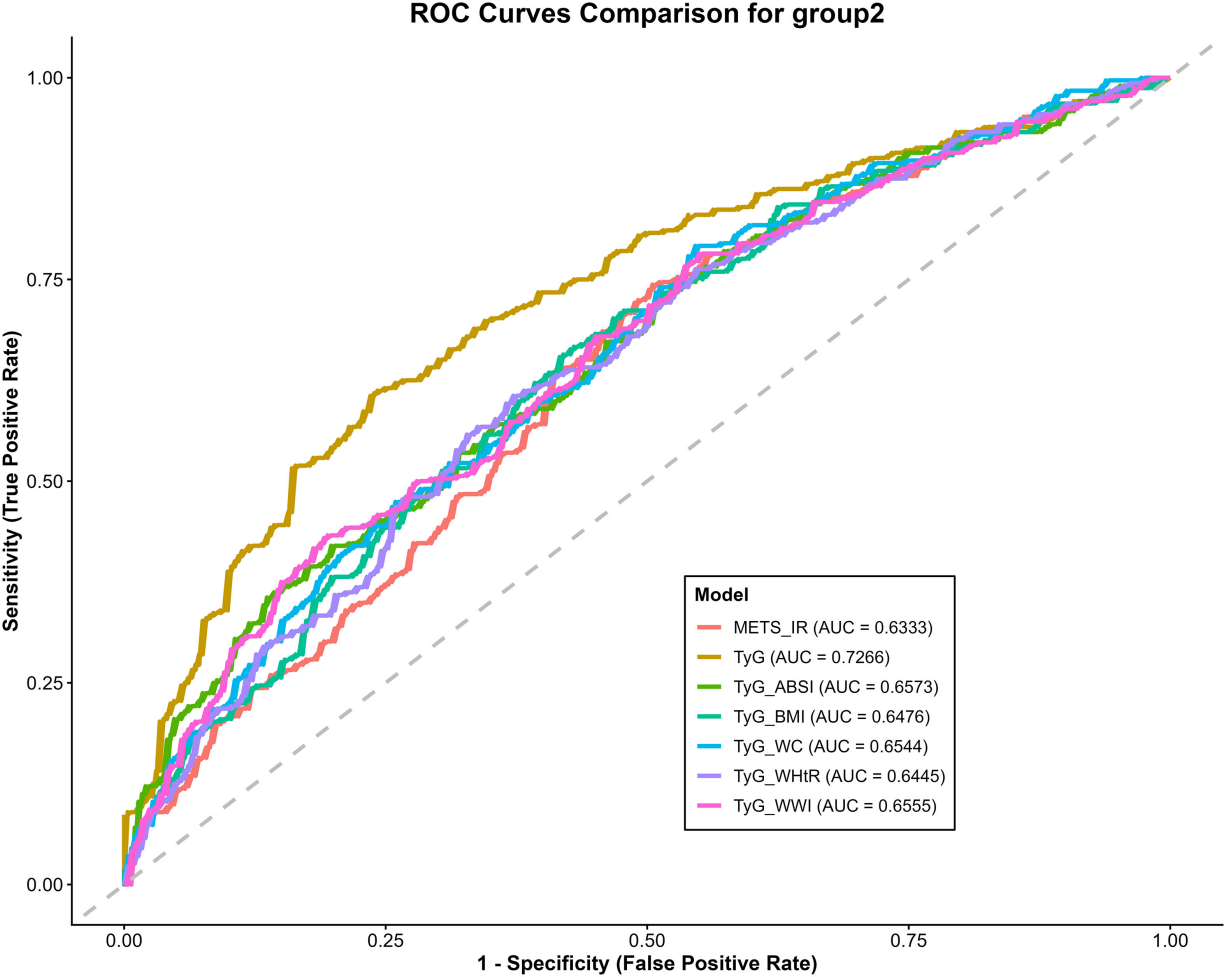
ROC cure graph for 7 insulin resistance surrogate indicators to discriminate the risk of co-occurrence of T2DM in community-dwelling older adults with MASLD.

The ROC curve analyses of the surrogate IR indexes for discriminating prevalent T2DM among community-dwelling older adults with MASLD are shown in **Figure 2** and **Table 3**. Overall, TyG showed the best performance, with an AUC of 0.726 (95% CI: 0.686-0.766). The optimal cut-off value was 9.225, with a sensitivity of 60.5%, a specificity of 76.4%, and a Youden index of 0.37. The four indexes with the highest AUCs were TyG, TyG-ABSI, TyG-WWI, and TyG-WC. TyG-WHtR, TyG-BMI, and METS-IR showed lower AUCs.

**Table 3:** Predictive ability of 7 surrogate insulin resistance indices for type 2 diabetes mellitus in community-dwelling older adults with MASLD.

Indices	AUC(95%CI)	Cut-off	Sen	Spe	Youden’s index	PLR	NLR	PPV	NPV
TyG	0.726 (0.686, 0.766)	9.225	0.605	0.764	0.370	2.56	0.52	43.6%	86.4%
TyG-ABSI	0.657 (0.614, 0.700)	7.584	0.419	0.802	0.221	2.12	0.72	38.9%	82.2%
TyG-WWI	0.655 (0.612, 0.698)	102.270	0.429	0.808	0.238	2.23	0.71	40.2%	82.4%
TyG-WC	0.654 (0.611, 0.697)	765.311	0.791	0.453	0.245	1.45	0.46	30.4%	87.5%
TyG-WHtR	0.644 (0.601, 0.688)	4.975	0.605	0.628	0.233	1.63	0.63	32.9%	83.8%
TyG-BMI	0.647 (0.604, 0.691)	231.601	0.653	0.583	0.237	1.57	0.59	32.1%	84.7%
METS-IR	0.633 (0.589, 0.677)	37.357	0.743	0.494	0.238	1.47	0.52	30.7%	85.9%

AUC, area under the receiver operating characteristic curve; CI, confidence interval. The optimal cutoff value was determined by maximizing Youden’s index (sensitivity + specificity - 1). Positive predictive value (PPV) and negative predictive value (NPV) were calculated based on the actual prevalence of type 2 diabetes mellitus in this study cohort (23.20%). All global models for discriminating type 2 diabetes mellitus were statistically significant (P < 0.05). Sen, sensitivity; Spe, specificity; PLR, positive likelihood ratio; NLR, negative likelihood ratio; PPV, positive predictive value; NPV, negative predictive value.

The DeLong test showed that TyG had a higher AUC than the other indexes. After Bonferroni correction, the AUC of TyG remained significantly greater than those of TyG-ABSI, TyG-WWI, TyG-WC, and TyG-WHtR. Based on the overall comparisons, TyG, TyG-ABSI, TyG-WWI, and TyG-WC were selected as the key indexes for subsequent RCS and exploratory model-discrimination analyses. The complete pairwise comparisons based on the DeLong test are presented in **Supplementary Table 3**.

### 3.5 Overall and nonlinear associations of TyG-related indexes with prevalent T2DM among participants with MASLD

**Figure 3:**
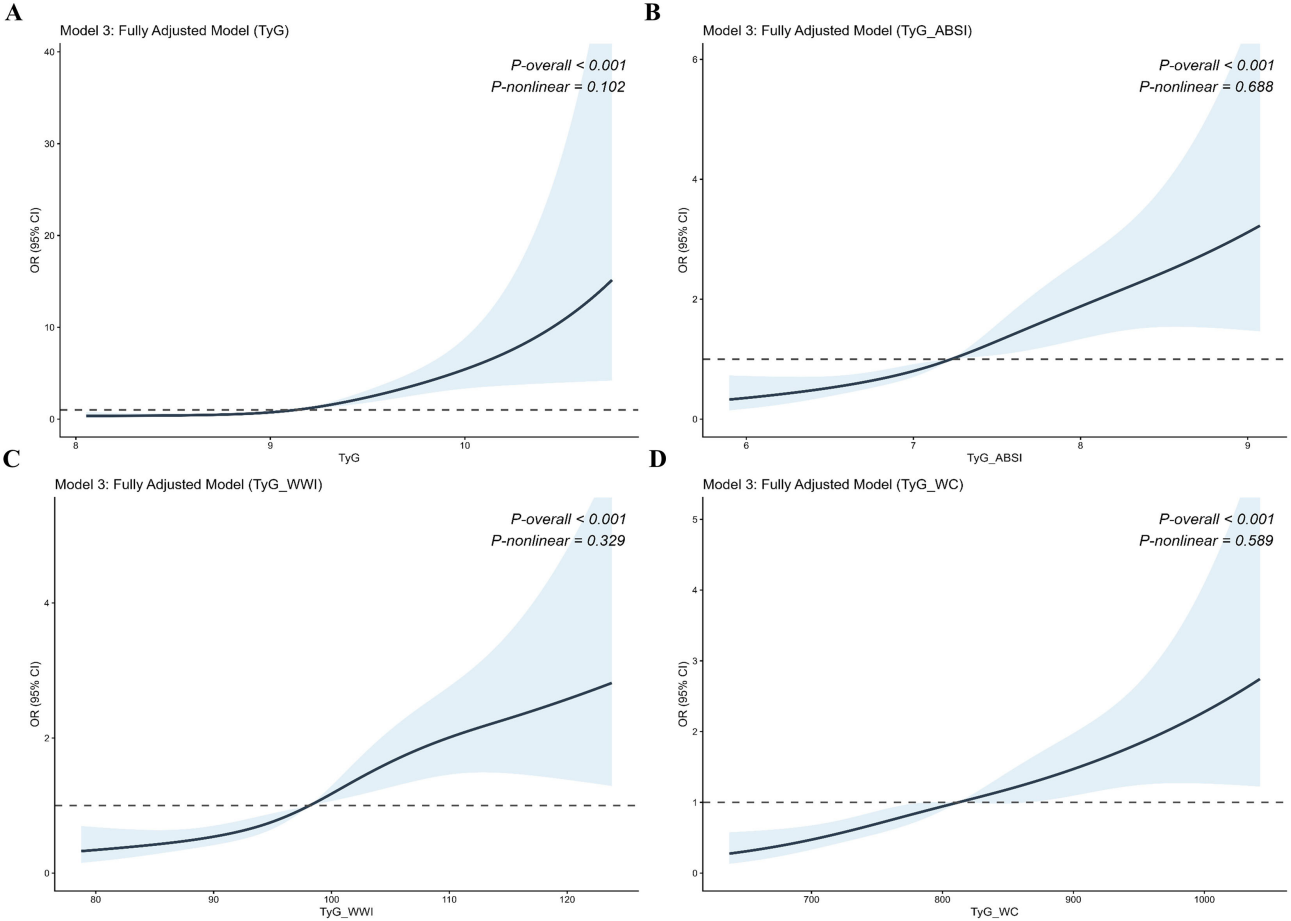
The RCS curves representing the dose-response relationships of the top 4 surrogate indicators of insulin resistance. **(A)** TyG, **(B)** TyG-ABSI, **(C)** TyG-WWI, **(D)** TyG-WC. Adjusted Model: Gender, Age, Marital, Education, Alcohol, Smoke, Hypertension, Exercise, ALT, AST, TC, LDL, Cr.

The RCS analyses of TyG, TyG-ABSI, TyG-WWI, and TyG-WC with prevalent T2DM among participants with MASLD are shown in **Figure 3**. All four indexes were significantly associated with prevalent T2DM overall (all P-overall < 0.001). In the fully adjusted model, the spline curves suggested a generally increasing probability of prevalent T2DM with higher levels of these indexes. However, the tests for nonlinearity were not statistically significant (TyG: P-nonlinear = 0.102; TyG-ABSI: P-nonlinear = 0.688; TyG-WWI: P-nonlinear = 0.329, TyG-WC: P-nonlinear = 0.589);, indicating linear or monotonic overall associations. Consequently, no threshold or inflection point analyses were performed. The results of Models 1 and 2 are presented in **Supplementary Figures 6**–**S9** and were generally consistent with those of the fully adjusted model.

### 3.6 Subgroup analyses of the associations of TyG-related indexes with T2DM among participants with MASLD

**Figure 4:**
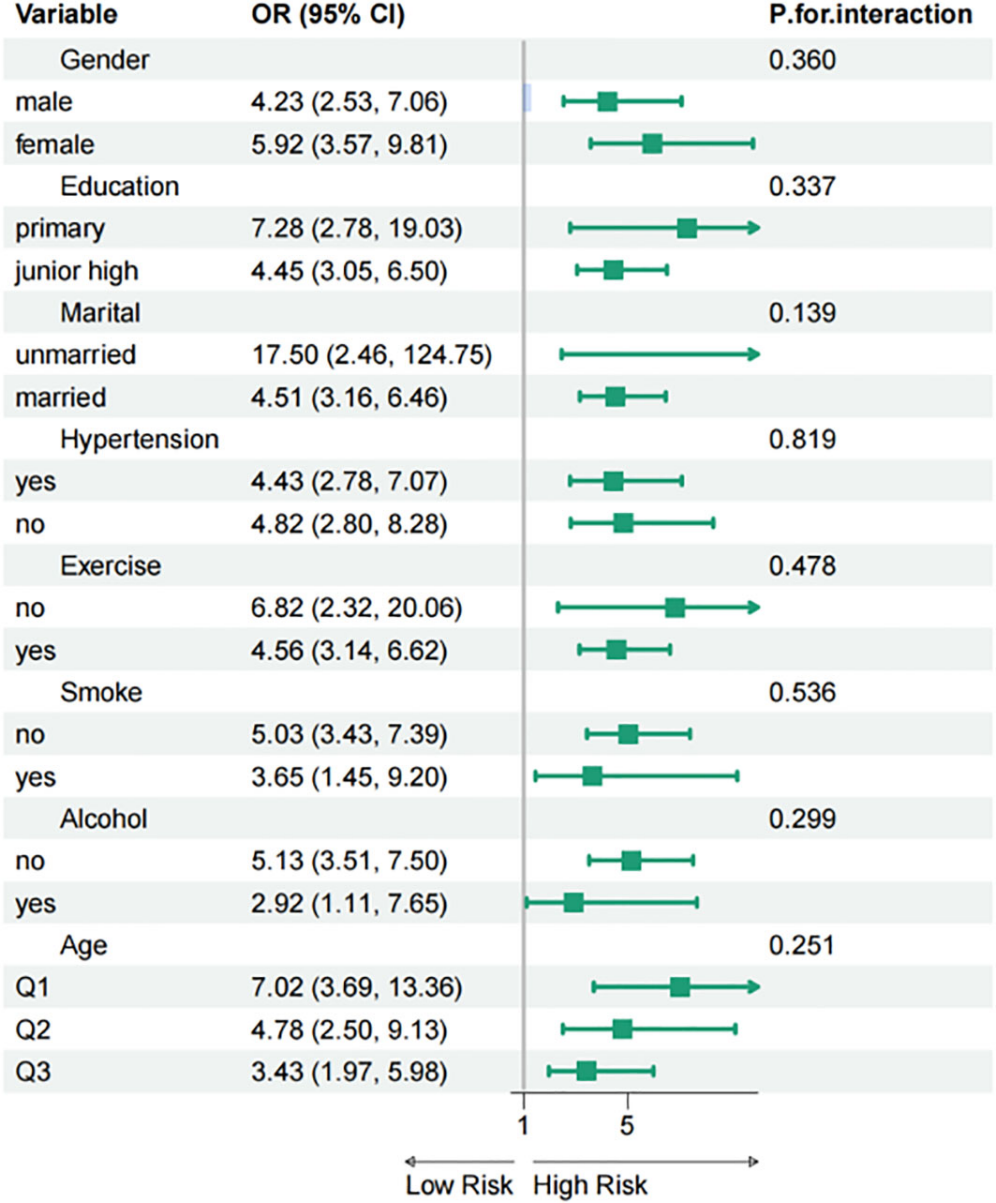
Subgroup analysis of the association between TyG index and T2DM in patients with MASLD.

The subgroup analysis of the association between TyG and T2DM among community-dwelling older adults with MASLD is shown in **Figure 4**. Higher TyG was significantly associated with an increased risk of T2DM, with an overall OR of 5.38 (95% CI: 3.70–7.83). Stratified analyses showed that this association was consistent across subgroups defined by age, gender, educational level, marital status, physical exercise, smoking, alcohol drinking, and history of hypertension. In all subgroups, higher TyG was associated with a higher risk of T2DM.

No significant interactions were observed for age, gender, educational level, marital status, physical exercise, smoking, alcohol drinking, or history of hypertension, suggesting that the association between TyG and T2DM was stable across subgroups. The subgroup analyses of the other three superior indexes, namely TyG-ABSI, TyG-WWI, and TyG-WC, yielded similar results, with no significant effect modification observed. Detailed results are presented in **Supplementary Figures 1**-**S3**.

### 3.7 Exploratory incremental discrimination of TyG-related indexes for prevalent T2DM among participants with MASLD

**Figure 5:**
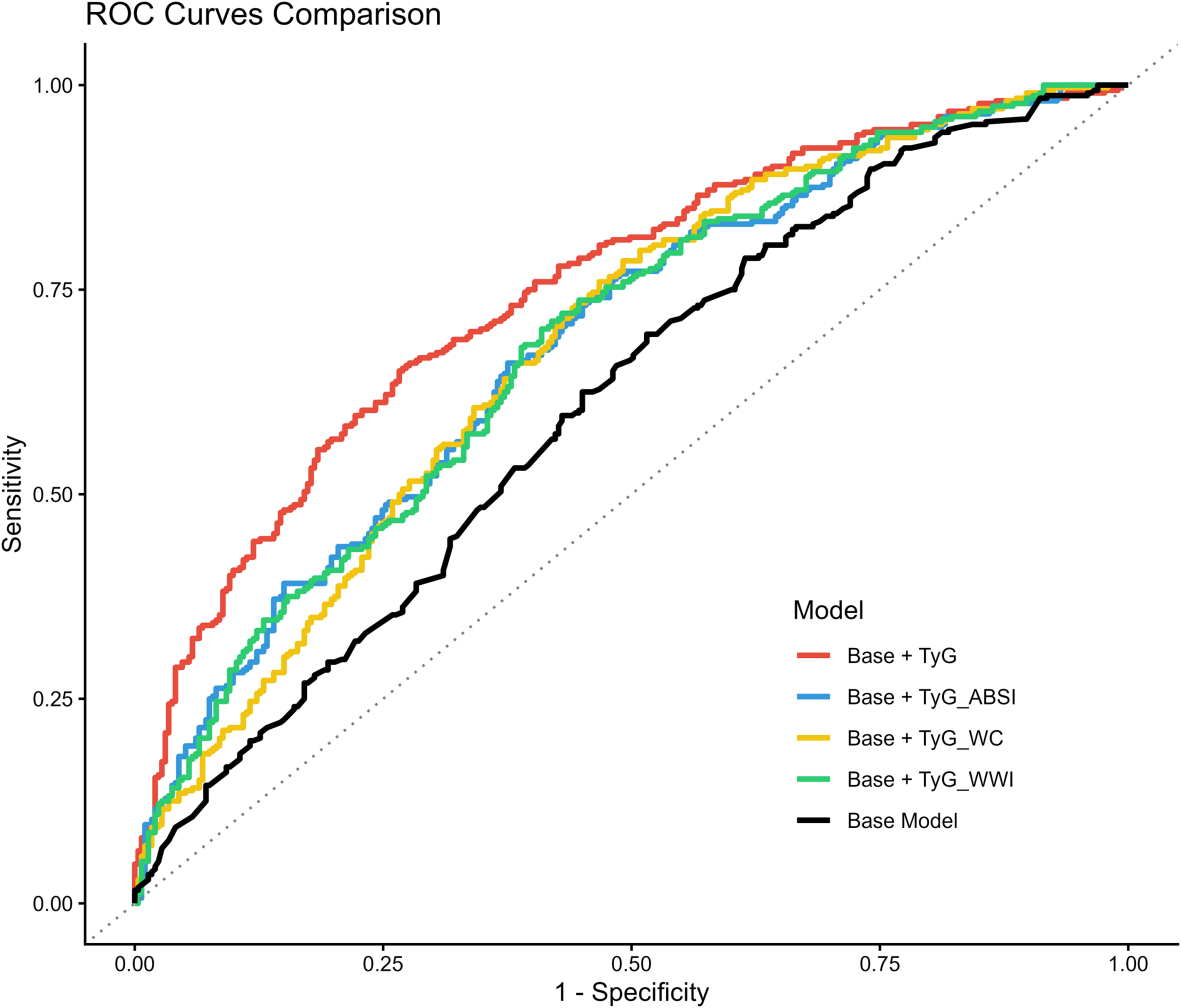
ROC curves of the basic model and the basic model + TyG related indicator model.

To evaluate the exploratory incremental value of the four top-performing TyG-related indexes, a basic model including age, gender, education, exercise, history of hypertension, and alcohol drinking was constructed on the basis of prior experience and the principle of practical availability. TyG, TyG-ABSI, TyG-WC, and TyG-WWI were then added separately to this basic model. The results are shown in **Figure 5**, **Table 4**, and **Supplementary Figures 4**, **5**.

**Table 4:** Comparison of discriminative capacity and probability agreement among different evaluation models.

Model	AUC (95% CI)	Δ AUC	*P* - value^a^	Brier score
Base Model	0.612(0.567,0.656)	–	–	0.238
Base + TyG	0.749(0.711,0.788)	0.138	< 0.001	0.203
Base + TyG-ABSI	0.686(0.644,0.728)	0.074	< 0.001	0.223
Base + TyG-WC	0.682(0.640,0.724)	0.070	< 0.001	0.224
Base + TyG-WWI	0.685(0.643,0.727)	0.073	< 0.001	0.223

AUC, area under the receiver operating characteristic curve; CI, confidence interval; TyG, triglyceride-glucose index. Base Model: Adjusted for age, gender, education, hypertension, exercise, and alcohol. ^a^ P for Δ AUC were calculated using DeLong’s test comparing the combined model with the base model. Brier Score ranges from 0 to 1, with lower values indicating closer agreement between predicted probabilities and actual outcomes. P<0.05 was considered statistically significant.

The basic model yielded an AUC of 0.612 (95% CI: 0.567–0.656). After adding each of the four indexes, the cross-sectional discrimination of the models improved significantly. TyG showed the most prominent incremental value, increasing the AUC to 0.749 (95% CI: 0.711–0.788), with a ΔAUC of 0.138 (P<0.001). In comparison, the models incorporating TyG-ABSI, TyG-WWI, and TyG-WC yielded lower AUCs of 0.686, 0.685, and 0.682, respectively, with ΔAUCs ranging from 0.070 to 0.074.

Furthermore, the Brier score decreased most substantially from the baseline level to 0.203 after the integration of TyG, indicating the best overall model probability agreement among the four candidates. Taken together, TyG demonstrated the most robust exploratory incremental discrimination and overall calibration performance.

Despite the outstanding incremental value demonstrated by TyG, these findings warrant cautious interpretation due to the inherent limitations of our cross-sectional design, which precludes the establishment of temporal or causal relationships. Therefore, future prospective cohort studies are highly warranted to validate the prospective predictive efficacy of TyG for T2DM development specifically among individuals with MASLD.

### 3.8 Sensitivity analysis

We conducted two sensitivity analyses to evaluate the robustness of our primary findings.

#### 3.8.1 Missing data analysis

Given that a substantial number of participants with missing key variables were excluded during the screening process, we compared the baseline demographic and lifestyle characteristics between the complete-case group and the missing-data group to assess the robustness of our primary findings and evaluate the potential impact of selection bias. The results showed that the two groups were generally well-balanced across most variables (all P > 0.05). Although age showed a statistically significant difference (P = 0.001, SMD = 0.154), the absolute difference was clinically negligible (1 year). Given that age was already adjusted for as a covariate in our primary regression model (Model 3), we concluded that the potential bias introduced by complete-case analysis is likely to be minimal. Detailed results are provided in **Supplementary Table 5**.

#### 3.8.2 Mathematical coupling analysis

To address the inherent mathematical coupling between TyG-related indices (which contain FPG) and the diagnosis of T2DM (which is partially defined by FPG), two additional sensitivity analyses were performed.

First, we excluded 66 participants whose T2DM status was defined solely by the FPG criterion (FPG ≥ 7.0 mmol/L without a prior diagnosis of T2DM or use of glucose-lowering medication). After exclusion, the restricted sample comprised 539 participants (293 MASLD-only and 246 with confirmed T2DM). In the fully adjusted model (Model 3), TyG, TyG-BMI, TyG-WC, TyG-WHtR, TyG-WWI, TyG-ABSI, and METS-IR remained significantly associated with T2DM. The discriminatory ability of all surrogate indices was attenuated compared to the primary analysis, but TyG still exhibited the highest AUC (0.7065, 95% CI: 0.6622-0.7509), confirming its superior performance.

Second, we adopted a more stringent definition by reclassifying those 66 participants with isolated FPG elevation into the non-T2DM group, resulting in a sample of 605 participants (359 non-T2DM and 246 with confirmed T2DM). Under this most stringent condition, only the four TyG-related indices (TyG, TyG-ABSI, TyG-WWI, TyG-WC) remained significantly associated with T2DM in the fully adjusted model, while other surrogates lost significance. In ROC analysis, TyG again showed the best discrimination, with an AUC of 0.6534 (95% CI: 0.6087-0.6980).

These analyses confirm that while a portion of TyG’s performance is attributable to shared mathematical components, its superiority over other surrogate indices is robust and not merely a statistical artifact. Detailed results are provided in **Supplementary Tables 6**, **7**.

## 4 Discussion

In the present study, based on a community-based health examination cohort of older adults in northwestern China, we systematically compared 12 surrogate IR indexes in relation to prevalent T2DM among participants with MASLD. Several main findings emerged. First, TyG, TyG-BMI, TyG-WC, TyG-WHtR, TyG-WWI, TyG-ABSI, and METS-IR were significantly and positively associated with prevalent T2DM among participants with MASLD. Second, TyG, TyG-ABSI, TyG-WWI, and TyG-WC showed relatively better discriminatory performance, with TyG yielding the highest AUC. Third, RCS analyses supported significant overall associations but did not provide evidence of statistically significant nonlinearity. Finally, adding these indexes to a basic model improved internal discrimination for prevalent T2DM, with TyG showing the best performance. These findings suggest that simple and low-cost surrogate IR indexes, particularly TyG, are associated with co-prevalent T2DM in older adults with MASLD, while prospective studies are needed before they can be used for risk prediction.

IR is a key shared pathophysiological basis of both MASLD and T2DM. It promotes hepatic lipid accumulation and lipotoxicity, impairs peripheral glucose utilization, increases hepatic glucose output, and contributes to chronic low-grade inflammation, thereby driving the development and progression of both diseases (20, 21). Older adults are particularly prone to complex metabolic abnormalities because of age-related visceral fat redistribution, loss of muscle mass, reduced insulin sensitivity, and the coexistence of multiple chronic conditions (22, 23). In this context, TyG may be especially informative because it combines FPG and TG, two routine biochemical markers that reflect both glucose and lipid metabolic disturbances, which are central features of IR and major metabolic links between MASLD and T2DM (24). Compared with more complex composite indexes, TyG is easier to calculate, requires fewer parameters, and may therefore be more practical for community-based screening.

Previous studies have shown that TyG and related indexes are associated with T2DM, fatty liver disease, and other cardiometabolic abnormalities (25, 26). TyG has also been proposed as a surrogate marker of HOMA-IR and the hyperinsulinemic-euglycemic clamp and has been linked to diabetes and metabolic syndrome in longitudinal or diagnostic settings (27). Some studies have suggested that TyG-derived indexes incorporating anthropometric parameters, such as TyG-BMI, TyG-WC, or TyG-WHtR, may further improve the identification of metabolic abnormalities (28). However, in older adults, BMI may be a less reliable indicator of metabolic health because of the redistribution of fat from subcutaneous to visceral depots and the progressive loss of muscle mass (29, 30). This may partly explain why TyG-BMI did not show stronger discrimination in our study.

In contrast, the attenuation of CVAI, LAP, VAI, TG/HDL-C, and AIP after full adjustment suggests that some lipid- or adiposity-based composite measures may not provide independent information beyond routinely adjusted covariates in this specific older MASLD population. From a clinical standpoint, aging is characterized by profound changes in body composition, notably sarcopenia coupled with the redistribution of adipose tissue toward ectopic and visceral depots. Consequently, standard anthropometric/lipid composite formulations originally validated in younger or general cohorts may fail to accurately capture the specific metabolic-dysfunction phenotype of older individuals with MASLD. Furthermore, in older adults, MASLD progression is often driven by multifaceted, age-related pathways—such as chronic low-grade inflammation, mitochondrial dysfunction, and cellular senescence—rather than being solely dictated by conventional lipid accumulation and insulin resistance. Therefore, while these composite indicators are robust markers in simpler bivariate analyses, their predictive utility is largely redundant once conventional cardiometabolic risk factors are comprehensively accounted for in this older demographic.

Our finding that TyG outperformed more complex TyG-derived indexes may be explained by several factors. First, older adults with MASLD have distinct metabolic characteristics, including increased visceral adiposity, reduced muscle mass, and lower insulin sensitivity (31, 32). In this population, TyG alone may already capture IR-related metabolic disturbances sufficiently well, whereas additional anthropometric parameters may contribute limited extra information. Second, composite indexes may be more vulnerable to the accumulation of measurement error because they depend on multiple variables, which may reduce their stability in community-based screening settings (33, 34). Third, MASLD is characterized by hepatic fat accumulation and lipid metabolic disturbances. The TG component of TyG may directly reflect hepatic lipid metabolism, whereas FPG reflects glucose dysregulation related to IR; together, they may more directly capture the shared metabolic basis of MASLD and T2DM (35, 36). In addition, TyG has been reported to be associated with markers of atherosclerosis and vascular injury, suggesting that it may reflect broader IR-related metabolic damage (37, 38). Our subgroup analyses further showed that the association between TyG and T2DM remained stable across subgroups defined by age, gender, educational level, and other characteristics, supporting its applicability in older adults with MASLD.

Several limitations of this study should be acknowledged. First, the cross-sectional design precludes any inferences of temporality or causality among MASLD, surrogate IR indexes, and T2DM. The analyses concern prevalent comorbid T2DM, not incident T2DM, and our findings should not be interpreted as evidence of prospective prediction. Second, the study population was derived from a single community in northwestern China, which may limit the generalizability of our findings to other populations. Third, a fundamental methodological limitation is the inherent information overlap (mathematical coupling) between the TyG index—which contains FPG—and the diagnosis of T2DM, which is partially defined by FPG. This shared component can artificially inflate the apparent discriminatory performance of TyG and its derived indices. We conducted rigorous sensitivity analyses by excluding T2DM cases defined solely by the FPG criterion (n=66) and, in the most stringent analysis, reclassifying these cases as non-T2DM. These analyses confirmed that TyG remained the top-performing index, although its AUC decreased from 0.726 to 0.707 and 0.653, respectively. These results indicate that while a portion of TyG’s performance is attributable to mathematical coupling, its superiority over other surrogates is not merely a statistical artifact. Nevertheless, due to this unavoidable shared component, TyG should not be interpreted as an independent diagnostic test for T2DM in this cross-sectional dataset. Fourth, exploratory medication sensitivity analyses suggested that the associations of TyG-related indexes were attenuated after accounting for medication categories or excluding users of antidiabetic agents, indicating that medication use and prior disease status may influence the observed cross-sectional associations. Fifth, fatty liver was assessed by abdominal ultrasonography rather than liver biopsy, MRI-PDFF, or controlled attenuation parameter (CAP). This may lead to misclassification, especially for mild steatosis. Sixth, the positive predictive value (PPV) and negative predictive value (NPV) depend on disease prevalence. Therefore, the diagnostic operating characteristics observed in this high-prevalence MASLD subgroup (23.2%) should not be directly extrapolated to lower-prevalence screening settings.

## 5 Conclusion

Among community-dwelling older MASLD patients, TyG, TyG-ABSI, TyG-WWI, and TyG-WC were significantly associated with prevalent T2DM. TyG consistently showed the most robust discriminatory capacity in ROC and internal model analyses, supported by rigorous sensitivity analyses. However, due to the cross-sectional design and inevitable informational overlap with T2DM criteria, these findings only reflect cross-sectional association and discrimination for prevalent comorbidity. Nonetheless, TyG may serve as a simple, integrative metabolic warning marker for coexisting T2DM in this high-risk group; its predictive efficacy in prospective cohort studies designed to avoid tautology warrants further validation.

## Data Availability

The original contributions presented in the study are included in the article/**Supplementary Material**. Further inquiries can be directed to the corresponding authors.
