# Supplementary material for: Discriminating prevalent type 2 diabetes among community-dwelling older adults with metabolic dysfunction-associated steatotic liver disease: a comparative analysis of 12 insulin resistance surrogates
Source: Front Endocrinol (Lausanne). 2026 Jul 8;17:1846547. doi: 10.3389/fendo.2026.1846547 (PMC13388057; doi:10.3389/fendo.2026.1846547)

**Supplementary Figure: S1-S9**

**Subgroup analysis:**

| 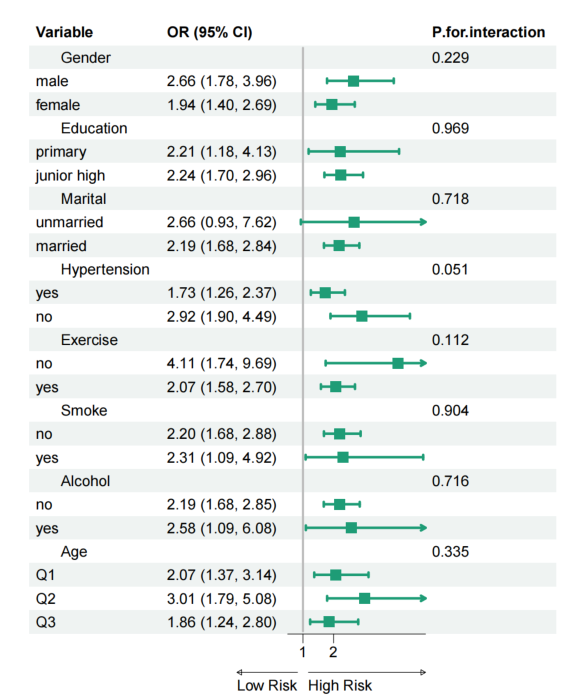 |
| --- |
| Supplement Figure 1 Subgroup analysis of the association between TyG-ABSI index and T2DM in patients with MAFLD. |

| 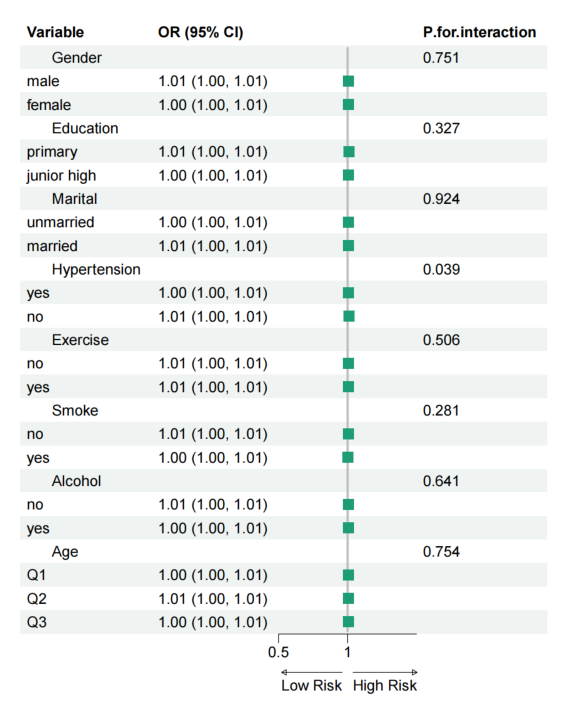 |
| --- |
| Supplement Figure 2 Subgroup analysis of the association between TyG-WC index and T2DM in patients with MAFLD. |

| 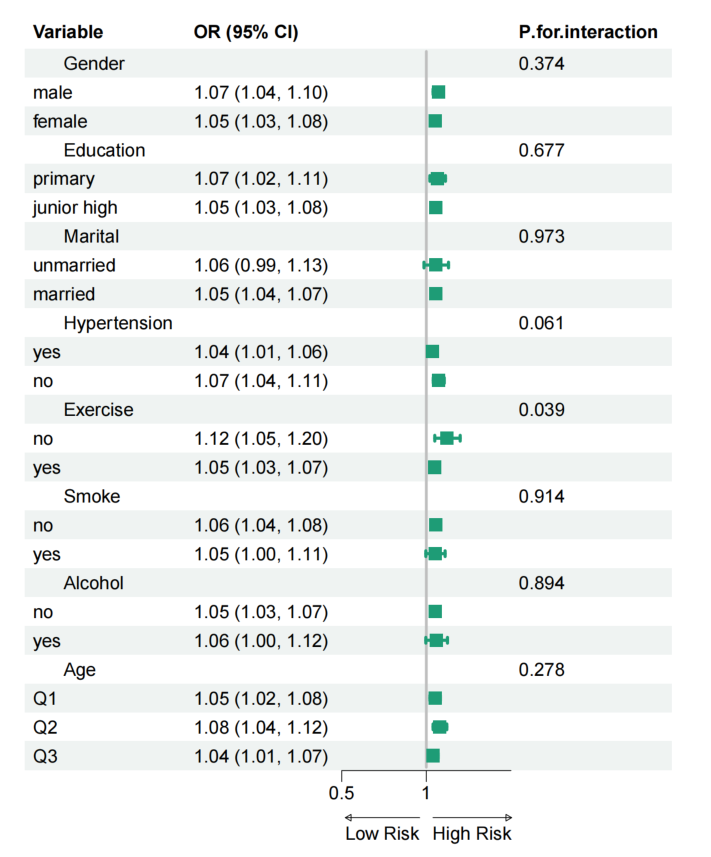 |
| --- |
| Supplement Figure 3 Subgroup analysis of the association between TyG-WWI index and T2DM in patients with MAFLD. |

**Calibration:**

| 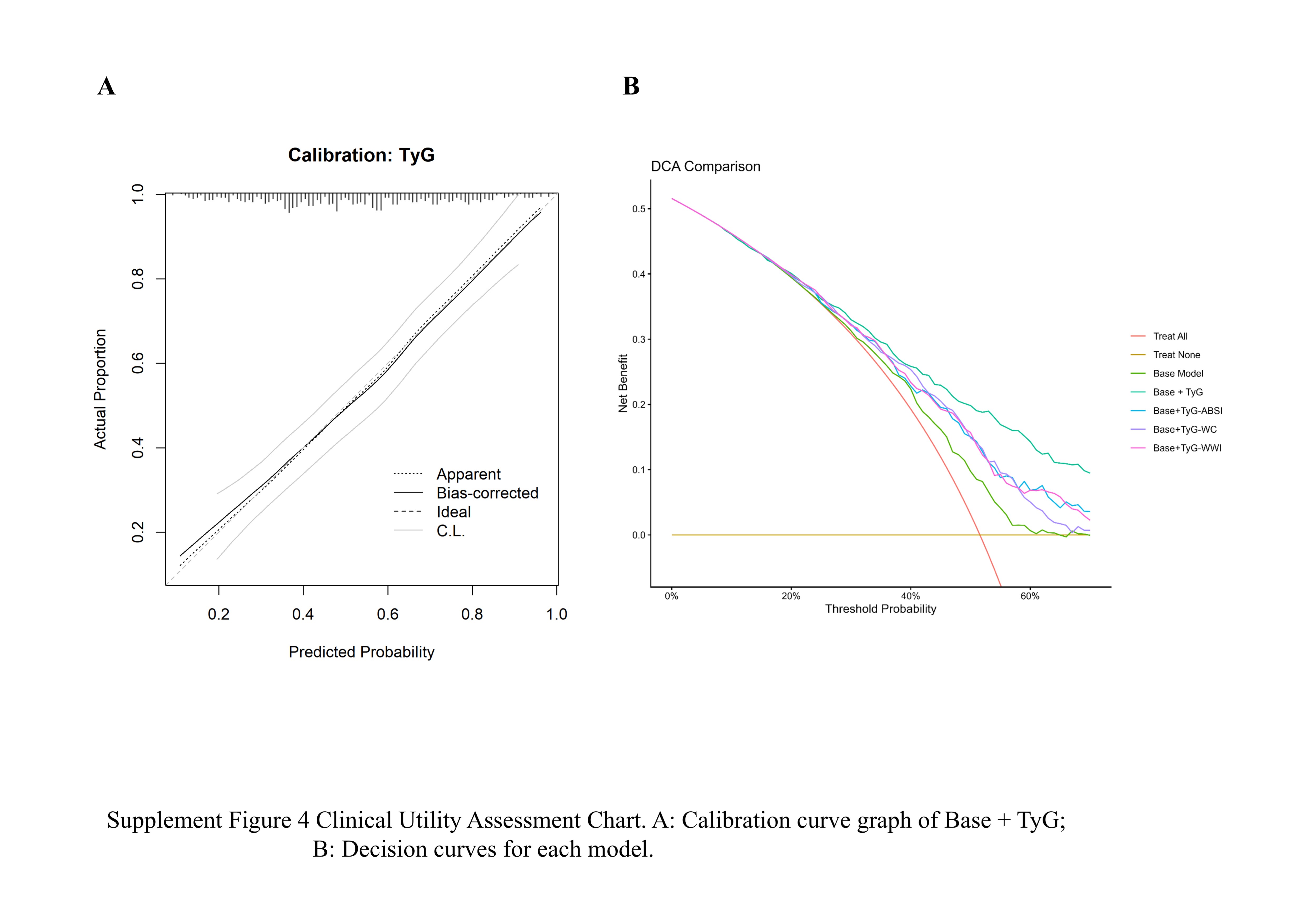 |
| --- |
| Supplement Figure 4 Clinical Utility Assessment Chart. A: Calibration curve graph of Base + TyG; B: Decision curves for each model. |

| 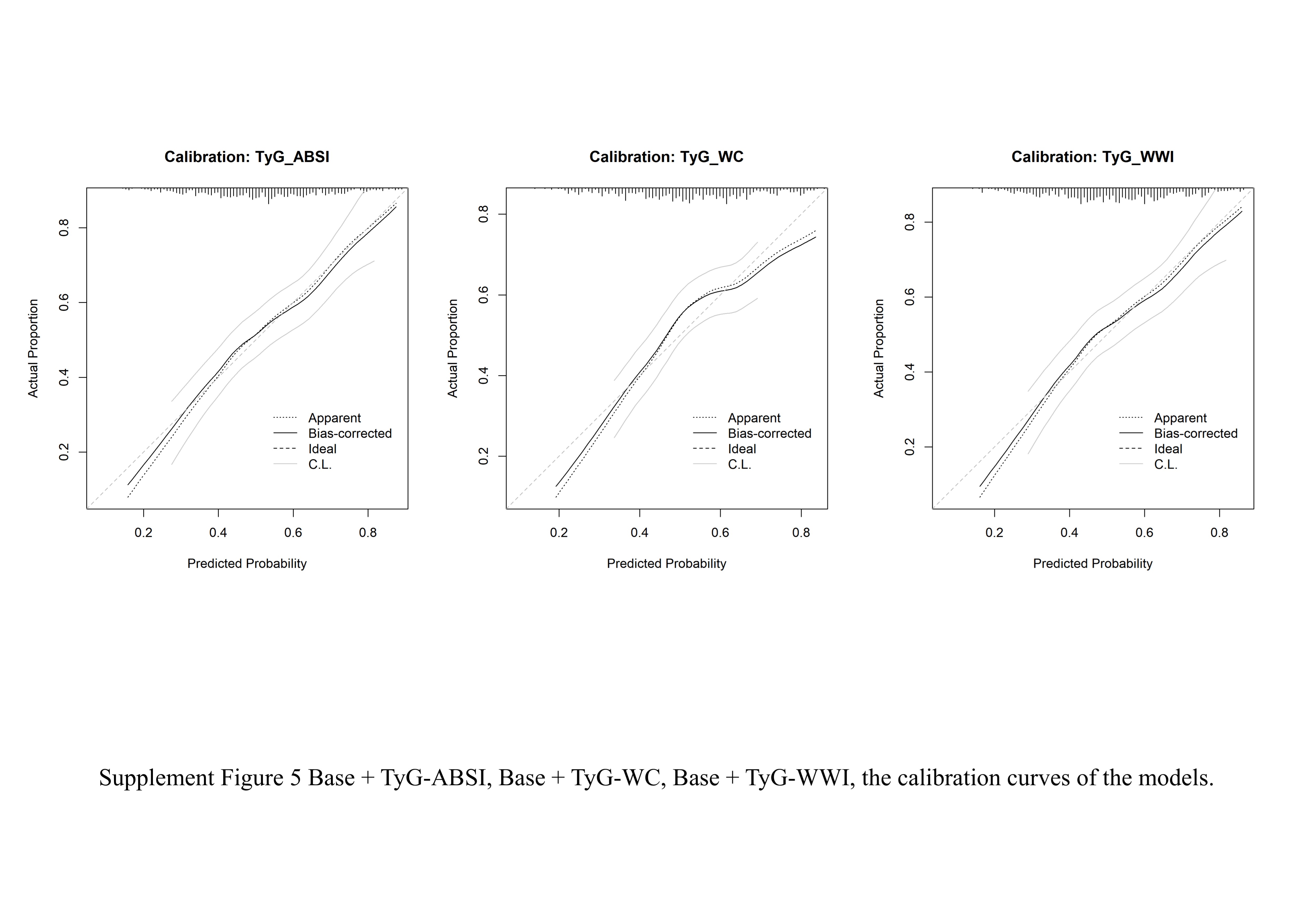 |
| --- |
| Supplement Figure 5 Base + TyG-ABSI, Base + TyG-WC, Base + TyG-WWI, the calibration curves of the models. |

**RCS curve:**


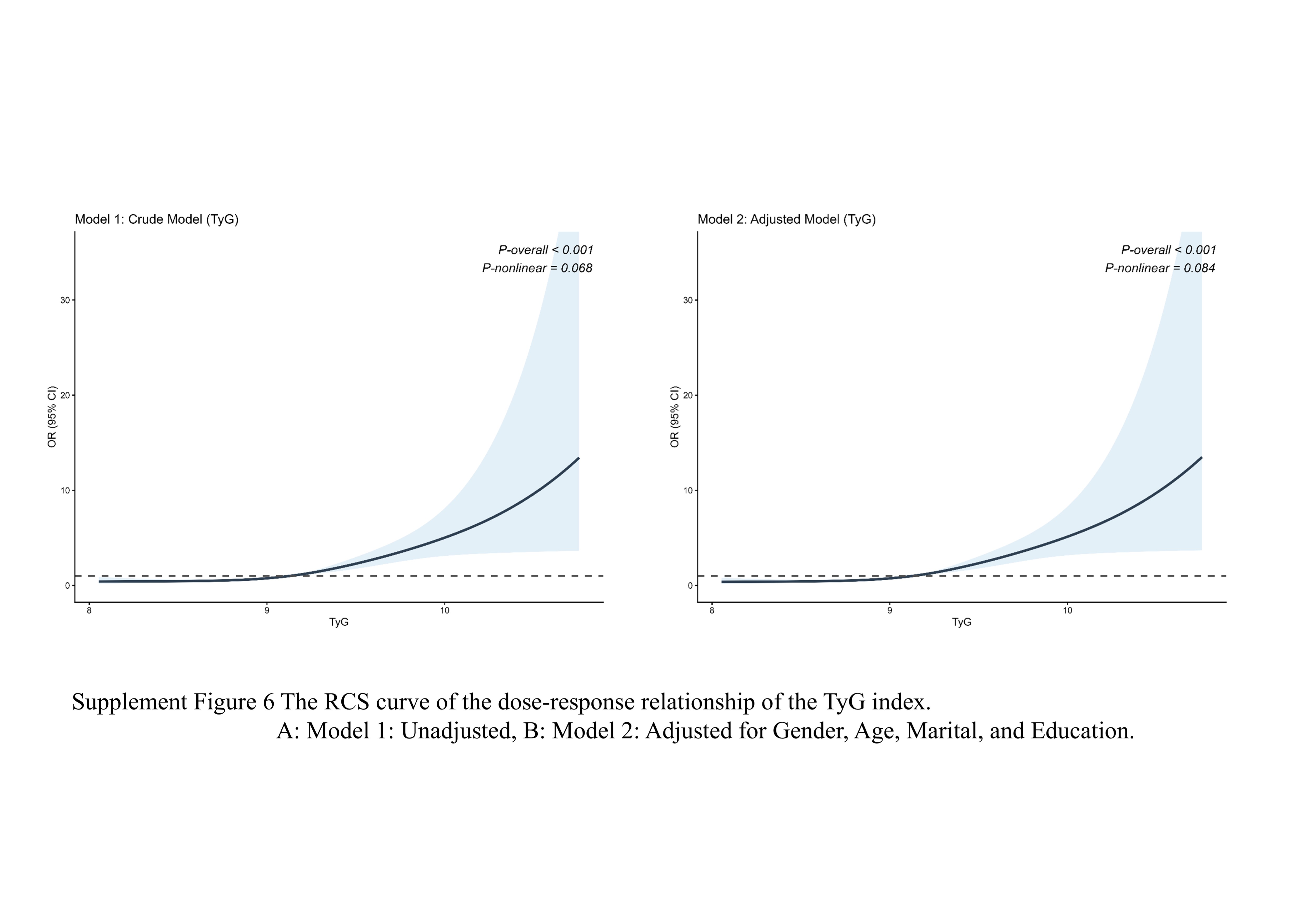


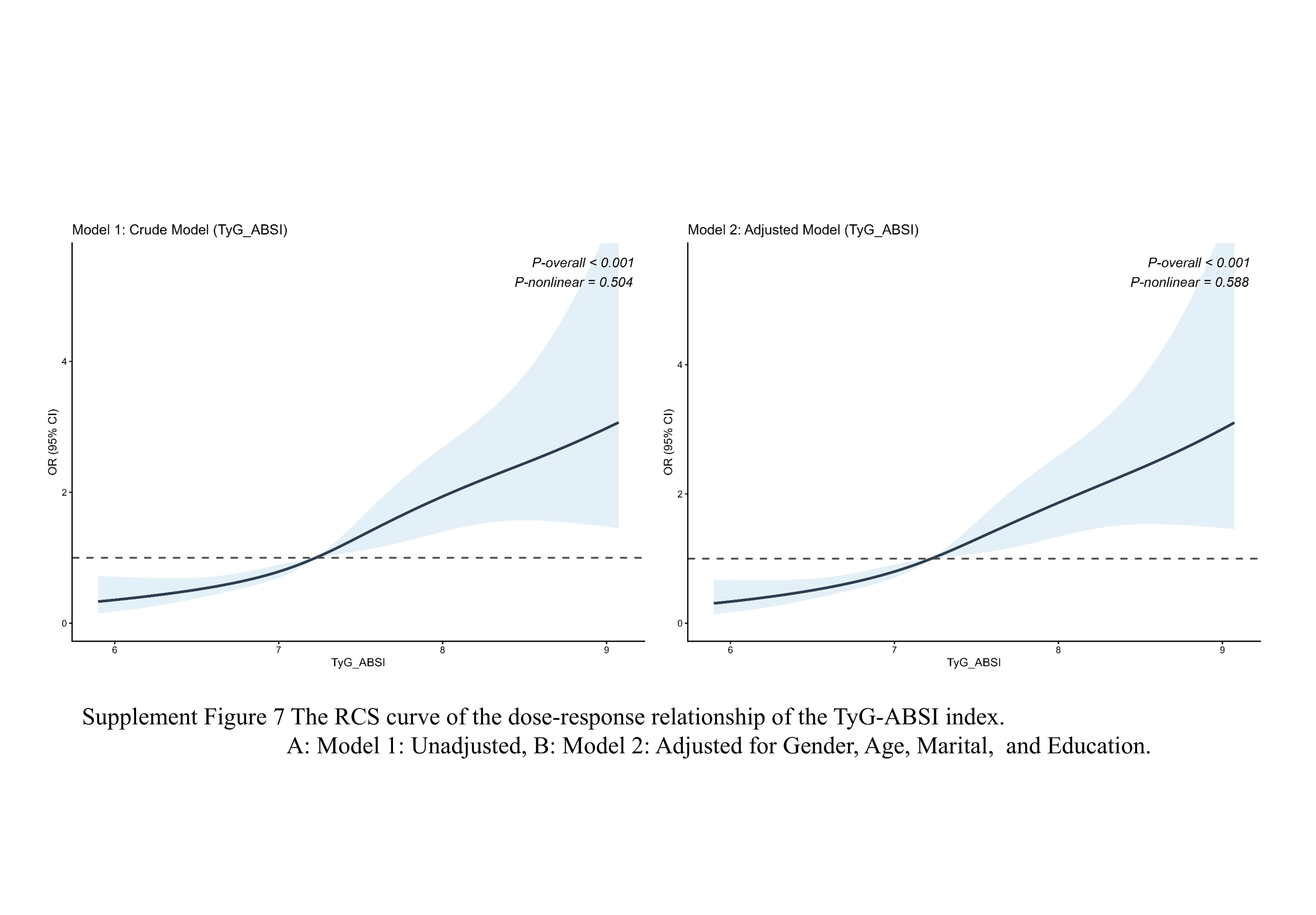


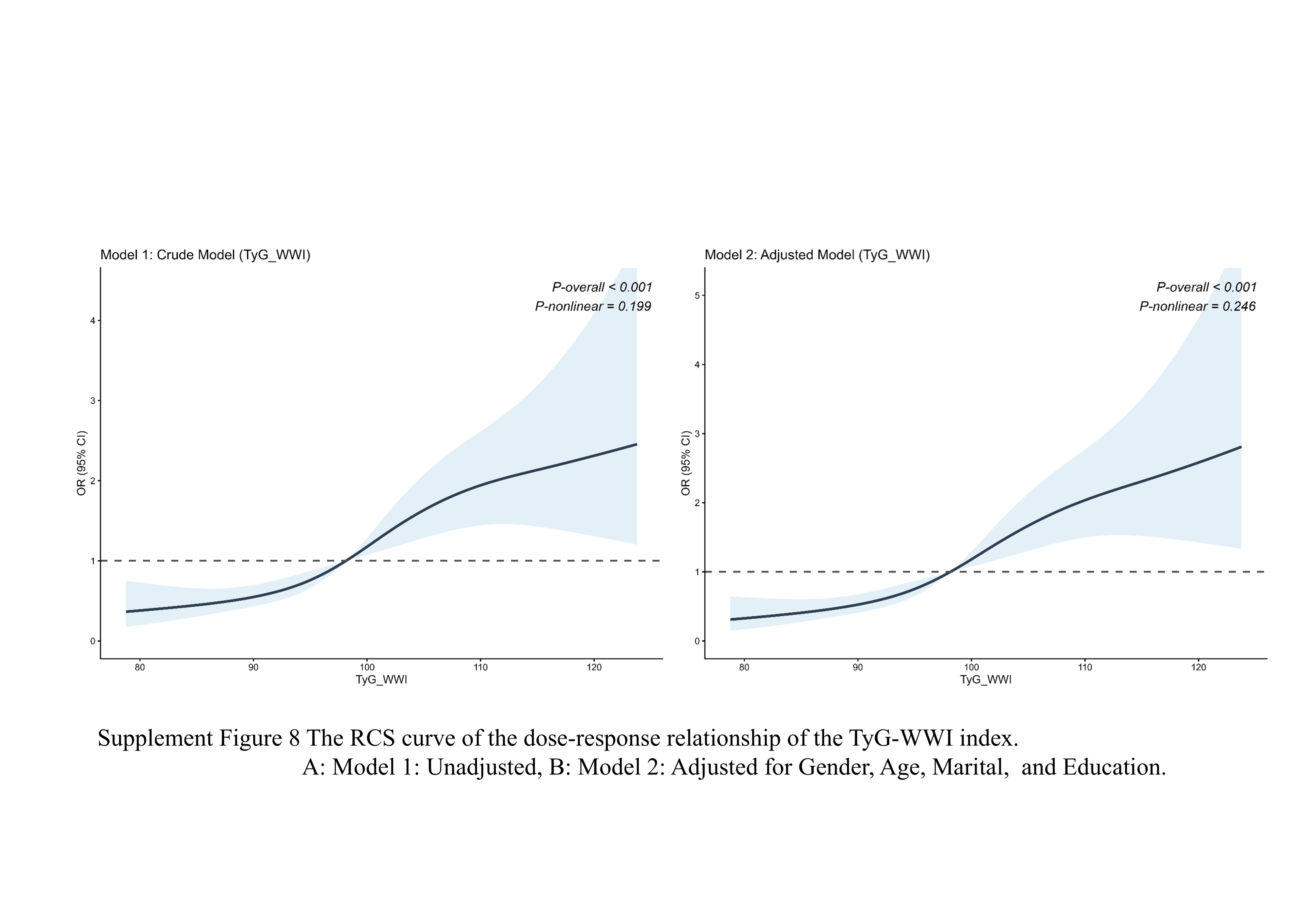


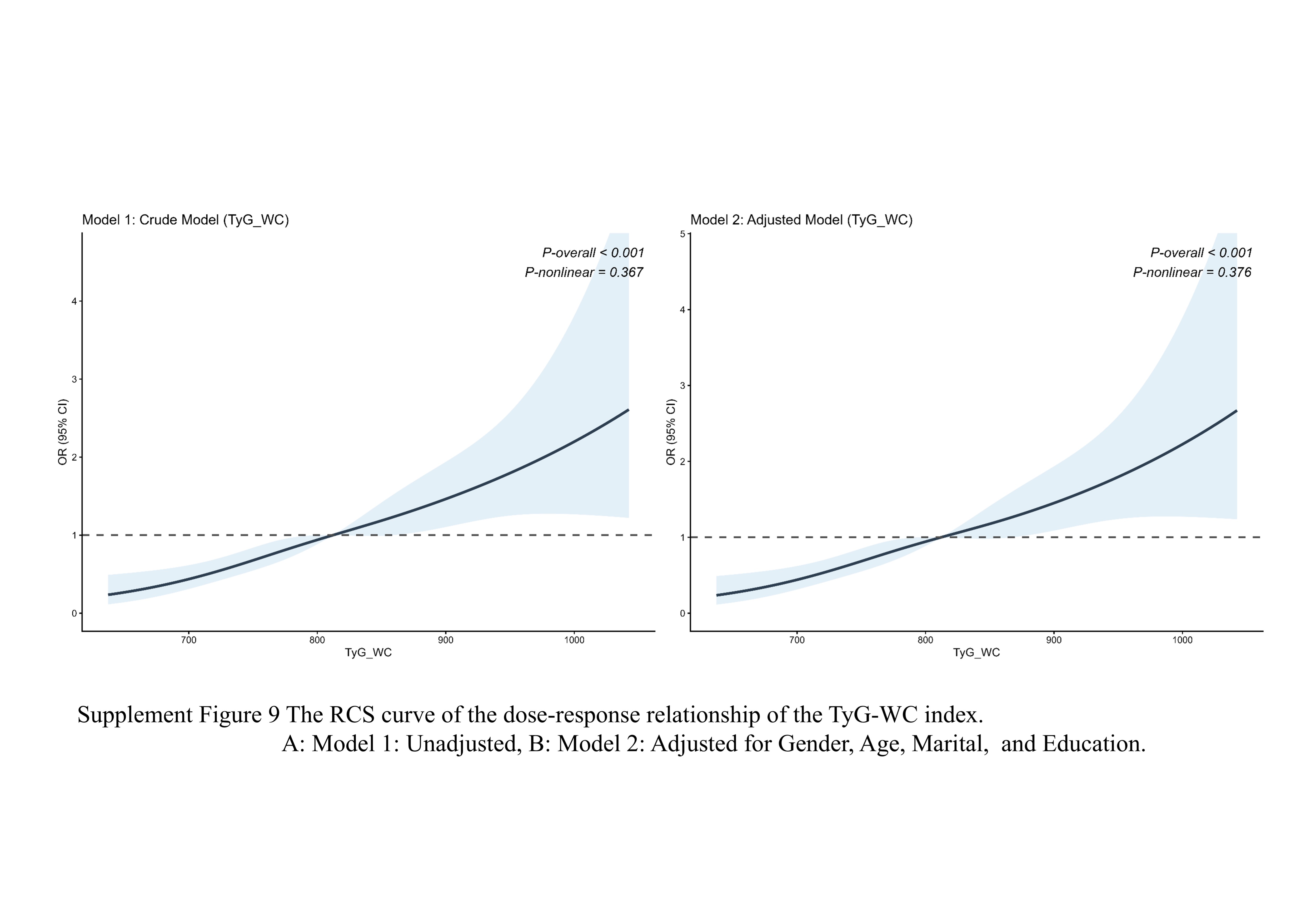


**Supplement Figure R1-C5b. Correlation heatmap**


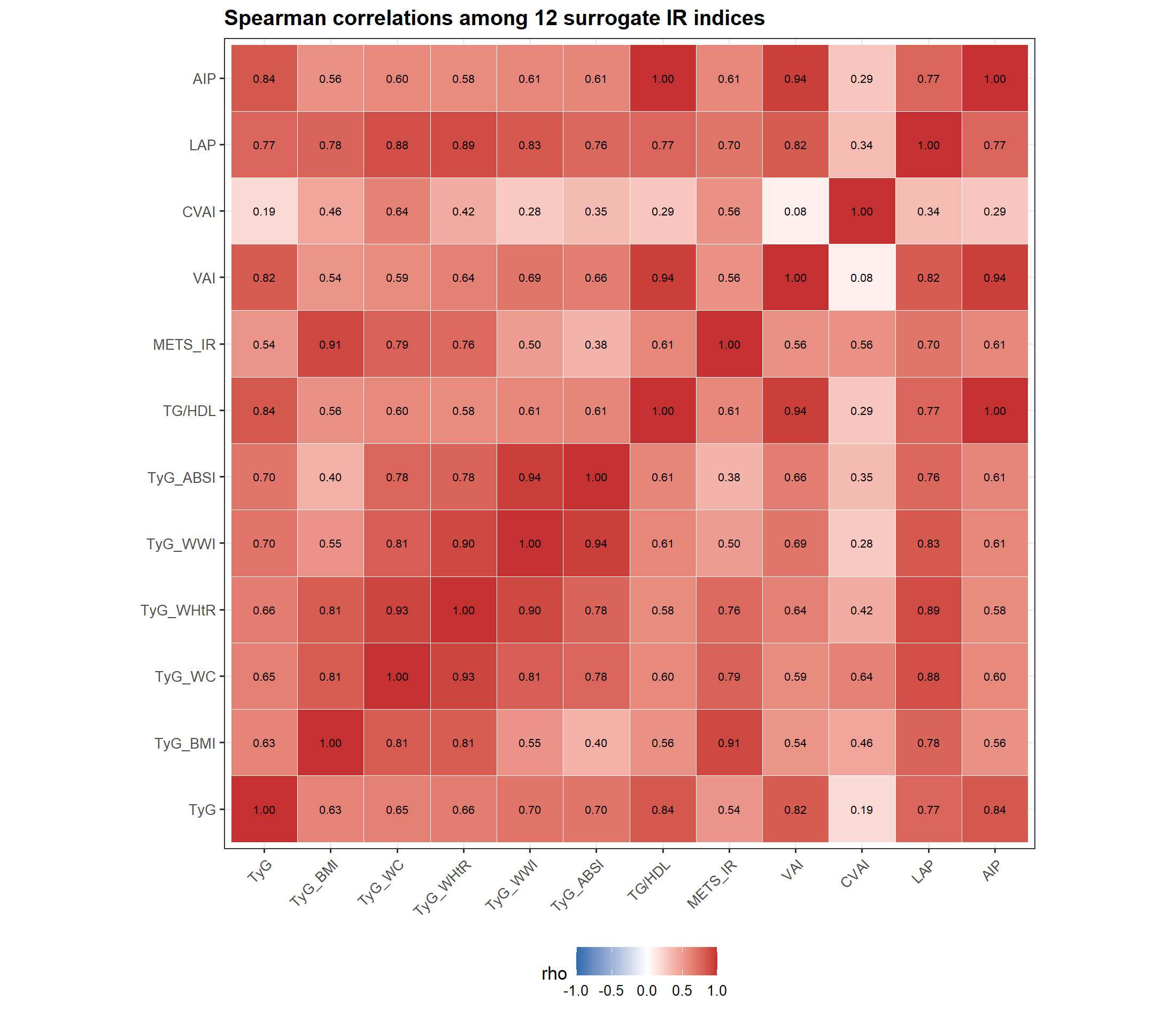

Supplement: Supplementary file 1 [file DataSheet1.docx]
