# Supplementary material for: Discriminating prevalent type 2 diabetes among community-dwelling older adults with metabolic dysfunction-associated steatotic liver disease: a comparative analysis of 12 insulin resistance surrogates
Source: Front Endocrinol (Lausanne). 2026 Jul 8;17:1846547. doi: 10.3389/fendo.2026.1846547 (PMC13388057; doi:10.3389/fendo.2026.1846547)
Supplement: Supplementary file 2 [file DataSheet2.docx]

**Supplementary Table: S1-S7**

| Supplementary Table S1. Formulas of the 12 surrogate insulin resistance indices | | |
| --- | --- | --- |
| **Abbreviation** | **Full name** | **Formula** |
| TyG | Triglyceride-glucose index | Ln [TG (mg/dL) × FPG (mg/dL) / 2] |
| TyG-BMI | TyG-body mass index | TyG × BMI |
| TyG-WC | TyG-waist circumference | TyG × WC (cm) |
| TyG-WHtR | TyG-waist-to-height ratio | TyG × WHtR |
| TyG-WWI | TyG-weight-adjusted-waist index | TyG × WWI |
| TyG-ABSI | TyG-a body shape index | TyG × ABSI |
| METS-IR | Metabolic score for insulin resistance | Ln [2 × FPG (mg/dL) + TG (mg/dL)] × BMI (kg/m²) / Ln [HDL-C (mg/dL)] |
| TG/HDL-C | Triglyceride-to-high-density lipoprotein cholesterol ratio | TG / HDL-C |
| VAI | Visceral adiposity index | Men: [WC / (39.68 + 1.88 × BMI)] × (TG / 1.03) × (1.31 / HDL-C); Women: [WC / (36.58 + 1.89 × BMI)] × (TG / 0.81) × (1.52 / HDL-C) |
| CVAI | Chinese visceral adiposity index | Men: -267.93 + 0.68 × age + 0.03 × BMI + 4.00 × WC (cm) + 22.00 × log10[TG (mmol/L)] − 16.32 × HDL-C (mmol/L); Women: -187.32 + 1.71 × age + 4.23 × BMI + 1.12 × WC (cm) + 39.76 × log10[TG (mmol/L)] − 11.66 × HDL-C (mmol/L) |
| LAP | Lipid accumulation product | Men: [WC (cm) − 65] × TG (mmol/L); Women: [WC (cm) − 58] × TG (mmol/L) |
| AIP | Atherogenic index of plasma | log10 [TG / HDL-C] |
| Note: Abbreviations: TyG, triglyceride-glucose index; BMI, body mass index; WC, waist circumference; FPG, fasting plasma glucose; TG, triglycerides; HDL-C, high-density lipoprotein cholesterol; WHtR, waist-to-height ratio; WWI, weight-adjusted-waist index, calculated as WC (cm)/√weight (kg); ABSI, a body shape index, calculated as WC (m)/[BMI^(2/3) × height^(1/2)]. When TG, FPG, and HDL-C are required in mg/dL, standard unit conversion was applied if the original measurements were in mmol/L: glucose, 1 mmol/L = 18 mg/dL; triglycerides, 1 mmol/L = 88.57 mg/dL; cholesterol, 1 mmol/L = 38.67 mg/dL. | | |

| Supplementary Table S2. Baseline characteristics of participants according to MAFLD and  type 2 diabetes mellitus status (n = 2641) | | | | | |  |
| --- | --- | --- | --- | --- | --- | --- |
| **Characteristics** | **Normal (N=1561)**^1^ | **T2DM (N=475)**^1^ | **MAFLD (N=293)**^1^ | **MAFLD＋T2DM (N=312)**^1^ | ***P***^2^ | ***SMD (Overall)*** |
| Age | 73.70 ± 7.17 | 74.23 ± 7.21 | 71.22 ± 5.42 | 71.83 ± 5.81 | ＜0.001 | 0.284 |
| Gender |  |  |  |  | 0.006 | 0.125 |
| Male | 700 (45%) | 249 (52%) | 119 (41%) | 145 (46%) |  |  |
| Female | 867 (55%) | 226 (48%) | 174 (59%) | 167 (54%) |  |  |
| Education |  |  |  |  | 0.278 | 0.075 |
| Primary | 272 (17%) | 90 (19%) | 54 (18%) | 43 (14%) |  |  |
| Junior high | 1,295 (83%) | 385 (81%) | 239 (82%) | 269 (86%) |  |  |
| Marital |  |  |  |  | 0.435 | 0.063 |
| Unmarried | 121 (7.7%) | 41 (8.6%) | 17 (5.8%) | 20 (6.4%) |  |  |
| Married | 1,446 (92%) | 434 (91%) | 276 (94%) | 292 (94%) |  |  |
| Exercise |  |  |  |  | 0.538 | 0.05 |
| No | 222 (14%) | 69 (15%) | 42 (14%) | 35 (11%) |  |  |
| Yes | 1,345 (86%) | 406 (85%) | 251 (86%) | 277 (89%) |  |  |
| Smoke |  |  |  |  | 0.775 | 0.026 |
| No | 1,343 (86%) | 400 (84%) | 246 (84%) | 264 (85%) |  |  |
| Yes | 224 (14%) | 75 (16%) | 47 (16%) | 48 (15%) |  |  |
| Alcohol |  |  |  |  | 0.203 | 0.066 |
| No | 1,443 (92%) | 434 (91%) | 260 (89%) | 280 (90%) |  |  |
| Yes | 124 (7.9%) | 41 (8.6%) | 33 (11%) | 32 (10%) |  |  |
| Hypertension |  |  |  |  | ＜0.001 | 0.27 |
| Yes | 722 (46%) | 317 (67%) | 152 (52%) | 210 (67%) |  |  |
| No | 845 (54%) | 158 (33%) | 141 (48%) | 102 (33%) |  |  |
| Drugs(Lowering blood glucose) |  |  |  |  | ＜0.001 | 1.48 |
| Yes | 0 (0%) | 377 (79%) | 0 (0%) | 142 (46%) |  |  |
| No | 1,567 (100%) | 98 (21%) | 293 (100%) | 170 (54%) |  |  |
| Drugs(Lowering Blood pressure) |  |  |  |  | ＜0.001 | 0.228 |
| Yes | 622 (40%) | 266 (56%) | 136 (46%) | 184 (59%) |  |  |
| No | 945 (60%) | 209 (44%) | 157 (54%) | 128 (41%) |  |  |
| Height (cm) | 162.13 ± 8.83 | 163.43 ± 8.53 | 162.75 ± 8.34 | 163.57 ± 8.80 | 0.015 | 0.096 |
| Weight (kg) | 60.85 ± 9.83 | 62.60 ± 9.37 | 68.23 ± 10.28 | 70.08 ± 11.11 | <0.001 | 0.545 |
| WC (cm) | 81.69 ± 9.14 | 83.88 ± 8.40 | 88.23 ± 9.88 | 89.62 ± 8.73 | <0.001 | 0.519 |
| BMI (kg/m^2^) | 23.10 ± 2.95 | 23.39 ± 2.76 | 25.70 ± 2.85 | 26.12 ± 3.02 | <0.001 | 0.654 |
| SBP (mmHg) | 138.17 ± 17.01 | 140.29 ± 16.10 | 140.70 ± 15.69 | 143.22 ± 15.89 | <0.001 | 0.16 |
| DBP (mmHg) | 76.33 ± 10.42 | 76.72 ± 10.23 | 77.33 ± 10.83 | 79.31 ± 10.33 | <0.001 | 0.153 |
| FPG (mmol/L) | 5.57 ± 1.18 | 8.03 ± 2.95 | 5.32 ± 0.48 | 8.04 ± 2.47 | <0.001 | 0.91 |
| TC (mmol/L) | 5.28 ± 1.39 | 5.19 ± 2.99 | 5.44 ± 1.32 | 5.56 ± 1.44 | <0.001 | 0.119 |
| TG (mmol/L) | 1.58 ± 0.93 | 1.67 ± 1.04 | 2.01 ± 1.01 | 2.30 ± 1.58 | <0.001 | 0.349 |
| HDL (mmol/L) | 1.51 ± 0.39 | 1.41 ± 0.36 | 1.44 ± 0.41 | 1.40 ± 0.32 | <0.001 | 0.157 |
| LDL (mmol/L) | 2.40 ± 0.80 | 2.29 ± 0.87 | 2.53 ± 0.81 | 2.45 ± 0.84 | <0.001 | 0.155 |
| ALT (U/L) | 23.00 ± 19.68 | 24.81 ± 26.39 | 27.77 ± 18.37 | 29.72 ± 19.69 | <0.001 | 0.185 |
| AST (U/L) | 24.27 ± 20.19 | 24.37 ± 23.18 | 25.67 ± 12.71 | 26.59 ± 13.40 | <0.001 | 0.08 |
| Cr (µmol/L) | 78.73 ± 39.09 | 80.01 ± 46.96 | 75.97 ± 20.25 | 77.12 ± 21.90 | 0.65 | 0.069 |
| TyG | 8.71 ± 0.55 | 9.08 ± 0.65 | 8.94 ± 0.48 | 9.40 ± 0.63 | <0.001 | 0.637 |
| TyG-BMI | 201.49 ± 30.66 | 212.76 ± 31.49 | 229.62 ± 28.10 | 245.58 ± 32.29 | <0.001 | 0.807 |
| TyG-WC | 712.26 ± 97.14 | 762.84 ± 103.05 | 788.68 ± 99.38 | 842.51 ± 97.81 | <0.001 | 0.702 |
| TyG-WHtR | 4.40 ± 0.62 | 4.68 ± 0.66 | 4.85 ± 0.62 | 5.16 ± 0.61 | <0.001 | 0.651 |
| TyG-WWI | 91.62 ± 10.14 | 96.71 ± 11.03 | 95.81 ± 10.43 | 101.08 ± 10.22 | <0.001 | 0.471 |
| TyG-ABSI | 6.91 ± 0.70 | 7.31 ± 0.78 | 7.11 ± 0.73 | 7.50 ± 0.73 | <0.001 | 0.451 |
| METS-IR | 33.45 ± 5.60 | 35.81 ± 5.69 | 38.09 ± 5.21 | 40.62 ± 5.41 | <0.001 | 0.723 |
| TG/HDL | 2.64 ± 1.94 | 2.98 ± 2.19 | 3.49 ± 2.22 | 4.09 ± 3.31 | <0.001 | 0.325 |
| AIP | -0.02 ± 0.27 | 0.03 ± 0.27 | 0.11 ± 0.25 | 0.16 ± 0.27 | <0.001 | 0.397 |
| CVAI | -659.78 ± 217.65 | -616.97 ± 208.06 | -595.06 ± 220.68 | -575.85 ± 170.32 | <0.001 | 0.224 |
| VAI | 4.25 ± 3.30 | 4.80 ± 3.97 | 5.82 ± 3.88 | 6.74 ± 5.97 | <0.001 | 0.321 |
| LAP | 33.65 ± 25.56 | 38.71 ± 31.62 | 55.38 ± 35.78 | 65.04 ± 47.70 | <0.001 | 0.511 |
| ^1^Mean ± SD; n (%)  ^2^Kruskal-Wallis rank sum test; Pearson's Chi-squared test  An SMD ≥ 0.1 indicates a meaningful clinical imbalance/difference between the four groups. | | | | | |  |

| Supplementary Table S3. Pairwise DeLong test comparisons of AUCs among the eight surrogate insulin resistance indices | | | | | | | |
| --- | --- | --- | --- | --- | --- | --- | --- |
| **Comparison** | **AUC_1** | **AUC_2** | **ΔAUC** | **Z statistic** | **P (raw)** | **Significant after Bonf.**  **（P＜0.0024）** | **Test** |
| TyG vs TyG_ABSI | 0.7266 | 0.6573 | 0.0693 | 3.7164 | 0.0002 | Yes | DeLong test |
| TyG vs TyG_WWI | 0.7266 | 0.6555 | 0.0710 | 3.7591 | 0.0002 | Yes | DeLong test |
| TyG vs TyG_WC | 0.7266 | 0.6544 | 0.0722 | 3.2915 | 0.0010 | Yes | DeLong test |
| TyG vs TyG_WHtR | 0.7266 | 0.6445 | 0.0821 | 3.8288 | 0.0001 | Yes | DeLong test |
| TyG vs TyG_BMI | 0.7266 | 0.6476 | 0.0790 | 3.4969 | 0.0005 | Yes | DeLong test |
| TyG vs METS_IR | 0.7266 | 0.6333 | 0.0933 | 3.8398 | 0.0001 | Yes | DeLong test |
| TyG_ABSI vs TyG_WWI | 0.6573 | 0.6555 | 0.0018 | 0.2107 | 0.8331 | No | DeLong test |
| TyG_ABSI vs TyG_WC | 0.6573 | 0.6544 | 0.0029 | 0.1793 | 0.8577 | No | DeLong test |
| TyG_ABSI vs TyG_WHtR | 0.6573 | 0.6445 | 0.0129 | 0.7776 | 0.4368 | No | DeLong test |
| TyG_ABSI vs TyG_BMI | 0.6573 | 0.6476 | 0.0097 | 0.3632 | 0.7165 | No | DeLong test |
| TyG_ABSI vs METS_IR | 0.6573 | 0.6333 | 0.0240 | 0.8944 | 0.3711 | No | DeLong test |
| TyG_WWI vs TyG_WC | 0.6555 | 0.6544 | 0.0011 | 0.0725 | 0.9422 | No | DeLong test |
| TyG_WWI vs TyG_WHtR | 0.6555 | 0.6445 | 0.0111 | 1.0031 | 0.3158 | No | DeLong test |
| TyG_WWI vs TyG_BMI | 0.6555 | 0.6476 | 0.0080 | 0.3357 | 0.7371 | No | DeLong test |
| TyG_WWI vs METS_IR | 0.6555 | 0.6333 | 0.0223 | 0.9045 | 0.3657 | No | DeLong test |
| TyG_WC vs TyG_WHtR | 0.6544 | 0.6445 | 0.0099 | 0.9474 | 0.3434 | No | DeLong test |
| TyG_WC vs TyG_BMI | 0.6544 | 0.6476 | 0.0068 | 0.4289 | 0.6680 | No | DeLong test |
| TyG_WC vs METS_IR | 0.6544 | 0.6333 | 0.0211 | 1.2776 | 0.2014 | No | DeLong test |
| TyG_WHtR vs TyG_BMI | 0.6445 | 0.6476 | -0.0031 | -0.2001 | 0.8414 | No | DeLong test |
| TyG_WHtR vs METS_IR | 0.6445 | 0.6333 | 0.0112 | 0.6369 | 0.5242 | No | DeLong test |
| TyG_BMI vs METS_IR | 0.6476 | 0.6333 | 0.0143 | 1.3654 | 0.1721 | No | DeLong test |
| Note: ΔAUC = AUC_1 − AUC_2. Differences between AUCs were compared using the DeLong test. The Bonferroni correction was applied to adjust for 28 pairwise comparisons within this table. | | | | | | | |

| Supplementary Table S4. Variance Inflation Factor (VIF) for Variables in the Final Logistic Regression Models | | | | | |
| --- | --- | --- | --- | --- | --- |
| **Model** | **Exposure Variable** | **Adjusted Variables** | **Maximum VIF** | **Minimum VIF** | **Diagnostic Conclusion** |
| 3 | TyG | Age, Gender, Education, Marital status, Smoking, Alcohol, Activity, Hypertension, ALT, AST, LDL-C, Cr | 3.193 | 1.030 | Acceptable |
| 3 | TyG-BMI | Age, Gender, Education, Marital status, Smoking, Alcohol, Activity, Hypertension, ALT, AST, LDL-C, Cr | 3.187 | 1.032 | Acceptable |
| 3 | TyG-WC | Age, Gender, Education, Marital status, Smoking, Alcohol, Activity, Hypertension, ALT, AST, LDL-C, Cr | 3.177 | 1.034 | Acceptable |
| 3 | TyG-WHtR | Age, Gender, Education, Marital status, Smoking, Alcohol, Activity, Hypertension, ALT, AST, LDL-C, Cr | 3.183 | 1.031 | Acceptable |
| 3 | TyG-WWI | Age, Gender, Education, Marital status, Smoking, Alcohol, Activity, Hypertension, ALT, AST, LDL-C, Cr | 3.179 | 1.030 | Acceptable |
| 3 | TyG-ABSI | Age, Gender, Education, Marital status, Smoking, Alcohol, Activity, Hypertension, ALT, AST, LDL-C, Cr | 3.174 | 1.030 | Acceptable |
| 3 | METS-IR | Age, Gender, Education, Marital status, Smoking, Alcohol, Activity, Hypertension, ALT, AST, LDL-C, Cr | 3.196 | 1.033 | Acceptable |
| 3 | TG/HDL | Age, Gender, Education, Marital status, Smoking, Alcohol, Activity, Hypertension, ALT, AST, LDL-C, Cr | 3.169 | 1.031 | Acceptable |
| 3 | VAI | Age, Gender, Education, Marital status, Smoking, Alcohol, Activity, Hypertension, ALT, AST, LDL-C, Cr | 3.167 | 1.030 | Acceptable |
| 3 | CVAI | Age, Gender, Education, Marital status, Smoking, Alcohol, Activity, Hypertension, ALT, AST, LDL-C, Cr | 3.181 | 1.035 | Acceptable |
| 3 | LAP | Age, Gender, Education, Marital status, Smoking, Alcohol, Activity, Hypertension, ALT, AST, LDL-C, Cr | 3.169 | 1.033 | Acceptable |
| 3 | AIP | Age, Gender, Education, Marital status, Smoking, Alcohol, Activity, Hypertension, ALT, AST, LDL-C, Cr | 3.187 | 1.031 | Acceptable |
| Note: VIF < 5 indicates the absence of severe multicollinearity; Tolerance = 1/VIF. | | | | | |

| Supplementary Table S5. Comparison of Baseline Characteristics between Complete-Case Group and Missing-Data Group. | | | | |
| --- | --- | --- | --- | --- |
| **Variable** | **Complete-Case Group(n=2641)**^1^ | **Missing-Data Group(n=587)**^1^ | **P value**^2^ | **SMD** |
| Age | 73.29±6.918 | 74.32±6.438 | 0.001 | 0.154 |
| Gender |  |  | 0.108 | 0.076 |
| Male | 1211 (45.8) | 247 (42.1) |  |  |
| Female | 1430 (54.2) | 340 (57.9) |  |  |
| Education |  |  | 0.1 | 0.075 |
| Primary | 457 (17.3) | 119 (20.3) |  |  |
| Junior high | 2184 (82.7) | 468 (79.7) |  |  |
| Marital |  |  | 0.064 | 0.085 |
| Unmarried | 198 (7.5) | 58 (9.9) |  |  |
| Married | 2443 (92.5) | 529 (90.1) |  |  |
| Hypertension |  |  | 0.323 | 0.047 |
| yes | 1398 (53.0) | 297 (49.4) |  |  |
| no | 1243 (47.0) | 290 (49.4) |  |  |
| Diabetes |  |  | 0.751 | 0.017 |
| yes | 613 (23.2) | 132 (22.5) |  |  |
| no | 2028 (76.8) | 455 (77.5) |  |  |
| Exercise |  |  | 0.535 | 0.032 |
| no | 366 (13.9) | 75 (12.8) |  |  |
| yes | 2275 (86.1) | 512 (87.2) |  |  |
| Smoke |  |  | 0.679 | 0.022 |
| no | 2247 (85.1) | 504 (85.9) |  |  |
| yes | 394 (14.9) | 83 (14.1) |  |  |
| Alcohol |  |  | 1.0 | 0.001 |
| no | 2411 (91.3) | 536 (91.3) |  |  |
| yes | 230 (8.7) | 51 (8.7) |  |  |
| Height(cm) | 162.61±8.742 | 162.04±8.444 | 0.157 | 0.065 |
| Weight(kg) | 63.07±10.532 | 63.08±9.612 | 0.986 | 0.001 |
| BMI | 23.79±3.135 | 23.96±2.771 | 0.246 | 0.058 |
| WC(cm) | 83.74±9.522 | 84.44±7.992 | 0.097 | 0.08 |
| ^1^Mean ± SD; n (%)  ^2^Kruskal-Wallis rank sum test; Pearson's Chi-squared test  An SMD ≥ 0.1 indicates a meaningful clinical imbalance/difference between the two groups. | | | | |

| Supplementary Table S6. Sensitivity Analysis: Comparison of Discriminatory Ability of Surrogate IR Indices for T2DM Under Different T2DM Definitions | | | |
| --- | --- | --- | --- |
| Indices | Primary Analysis(n=605, Original T2DM definition) | Sensitivity Analysis 1 (n=539, Excluding FPG-only cases) | Sensitivity Analysis 2 (n=605, Reclassifying FPG-only cases) |
|  | AUC (95% CI) | AUC (95% CI) | AUC (95% CI) |
| **TyG** | **0.726 (0.686, 0.766)** | **0.7065 (0.6622, 0.7509)** | **0.6534 (0.6087, 0.6980)** |
| TyG-ABSI | 0.657 (0.614, 0.700) | 0.6421 (0.5956, 0.6887) | 0.6042 (0.5584, 0.6501) |
| TyG-WWI | 0.655 (0.612, 0.698) | 0.6379 (0.5911, 0.6847) | 0.5994 (0.5535, 0.6454) |
| TyG-WC | 0.654 (0.611, 0.697) | 0.6246 (0.5777, 0.6715) | 0.5723 (0.5266, 0.6181) |
| TyG-WHtR | 0.644 (0.601, 0.688) | 0.6187 (0.5715, 0.6659) | 0.5737 (0.5277, 0.6197) |
| TyG-BMI | 0.647 (0.604, 0.691) | 0.6152 (0.5678, 0.6625) | 0.5621 (0.5160, 0.6081) |
| METS-IR | 0.633 (0.589, 0.677) | 0.5985 (0.5507, 0.6462) | 0.5456 (0.4995, 0.5916) |

| Supplementary Table S7. Multivariable logistic regression of 12 surrogate IR indices with prevalent T2DM under different T2DM definitions (Model 3). | | | | | |
| --- | --- | --- | --- | --- | --- |
| **Indices** | **Q1 (ref)** | **Q2 OR (95% CI)** | **Q3 OR (95% CI)** | **Q4 OR (95% CI)** | **P for trend** |
| **(A) Sensitivity Analysis 1: Excluding FPG-only cases (n=539)** | | | | | |
| TyG | 1.0 | 1.454(0.843, 2.521) | 2.880(1.665, 5.050) | 7.960(4.480, 14.51) | <0.001 |
| TyG-BMI | 1.0 | 2.082(1.232, 3.552) | 2.504(1.470, 4.311) | 3.229(1.887, 5.606) | <0.001 |
| TyG-WC | 1.0 | 1.804(1.075, 3.049) | 1.699(1.008, 2.880) | 3.306(1.923, 5.759) | <0.001 |
| TyG-WHtR | 1.0 | 1.270(0.752, 2.151) | 1.982(1.174, 3.371) | 2.402(1.400, 4.160) | <0.001 |
| TyG-WWI | 1.0 | 1.685(0.998, 2.865) | 1.545(0.907, 2.647) | 1.545(0.907, 2.647) | <0.001 |
| TyG-ABSI | 1.0 | 2.013(1.197, 3.415) | 1.726(1.020, 2.942) | 4.397(2.572, 7.641) | <0.001 |
| METS-IR | 1.0 | 1.860(1.107, 3.150) | 2.722(1.619, 4.628) | 2.389(1.422, 4.053) | <0.001 |
| CVAI | 1.0 | 1.637(0.984, 2.739) | 1.586(0.942, 2.683) | 1.161(0.671, 2.019) | 0.642 |
| AIP | 1.0 | 1.073(0.649, 1.774) | 1.009(0.605, 1.683) | 1.282(0.769, 2.145) | 0.404 |
| TG/HDL | 1.0 | 1.073(0.649, 1.774) | 1.009(0.605, 1.683) | 1.282(0.769, 2.145) | 0.404 |
| VAI | 1.0 | 1.035(0.621, 1.724) | 1.035(0.621, 1.724) | 1.206(0.704, 2.072) | 0.602 |
| LAP | 1.0 | 1.153(0.687, 1.935) | 0.849(0.500, 1.437) | 1.394(0.819, 2.379) | 0.403 |
| **(B) Sensitivity Analysis 2: Reclassifying FPG-only cases as non-T2DM (n=605)** | | | | | |
| TyG | 1.0 | 1.513(0.909, 2.535) | 1.920(1.144, 3.250) | 4.066(2.448, 6.855) | <0.001 |
| TyG-BMI | 1.0 | 1.921(1.173, 3.171) | 1.953(1.187, 3.237) | 1.855(1.120, 3.095) | 0.028 |
| TyG-WC | 1.0 | 1.448(0.887, 2.372) | 1.568(0.957, 2.581) | 1.892(1.136, 3.174) | 0.017 |
| TyG-WHtR | 1.0 | 0.933(0.567, 1.534) | 1.629(1.000, 2.664) | 1.376(0.832, 2.282) | 0.059 |
| TyG-WWI | 1.0 | 1.385(0.848, 2.271) | 1.190(0.721, 1.969) | 2.710(1.635, 4.534) | <0.001 |
| TyG-ABSI | 1.0 | 1.590(0.973, 2.611) | 1.143(0.692, 1.893) | 2.913(1.776, 4.829) | <0.001 |
| METS-IR | 1.0 | 1.833(1.126, 3.004) | 2.135(1.308, 3.514) | 1.424(0.865, 2.354) | 0.151 |
| CVAI | 1.0 | 1.440(0.891, 2.336) | 1.243(0.759, 2.040) | 1.000(0.594, 1.685) | 0.853 |
| AIP | 1.0 | 0.931(0.576, 1.505) | 0.950(0.584, 1.543) | 1.109(0.683, 1.803) | 0.660 |
| TG/HDL | 1.0 | 0.931(0.576, 1.505) | 0.950(0.584, 1.543) | 1.109(0.683, 1.803) | 0.660 |
| VAI | 1.0 | 0.811(0.498, 1.316) | 0.772(0.468, 1.270) | 0.996(0.601, 1.650) | 0.973 |
| LAP | 1.0 | 1.074(0.660, 1.748) | 0.714(0.430, 1.179) | 1.191(0.722, 1.968) | 0.833 |
